# Two-dimensional covalent organic framework films prepared on various substrates through vapor induced conversion

**DOI:** 10.1038/s41467-022-29050-9

**Published:** 2022-03-17

**Authors:** Minghui Liu, Youxing Liu, Jichen Dong, Yichao Bai, Wenqiang Gao, Shengcong Shang, Xinyu Wang, Junhua Kuang, Changsheng Du, Ye Zou, Jianyi Chen, Yunqi Liu

**Affiliations:** 1 Beijing National Laboratory for Molecular Sciences, Key Laboratory of Organic Solids, Institute of Chemistry, Chinese Academy of Sciences, 100190 Beijing, PR China; 2 University of Chinese Academy of Sciences, 100049 Beijing, PR China

**Keywords:** Synthetic chemistry methodology, Electronic devices, Polymers

## Abstract

Covalent organic frameworks (COFs) can exhibit high specific surface area and catalytic activity, but traditional solution-based synthesis methods often lead to insoluble and infusible powders or fragile films on solution surface. Herein we report large-area –C=N– linked two-dimensional (2D) COF films with controllable thicknesses via vapor induced conversion in a chemical vapor deposition (CVD) system. The assembly process is achieved by reversible Schiff base polycondensation between PyTTA film and TPA vapor, which results in a uniform organic framework film directly on growth substrate, and is driven by π‐π stacking interactions with the aid of water and acetic acid. Wafer-scale 2D COF films with different structures have been successfully synthesized by adjusting their building blocks, suggesting its generic applicability. The carrier mobility of PyTTA-TPA COF films can reach 1.89 × 10^−3^ cm^2^ V^−1^ s^−1^. When employed as catalysts in hydrogen evolution reaction (HER), they show high electrocatalytic activity compared with metal-free COFs or even some metallic catalysts. Our results represent a versatile route for the direct construction of large-area uniform 2D COF films on substrates towards multi-functional applications of 2D π‐conjugated systems.

## Abstract (web summary)

Solution-based synthesis of covalent organic frameworks (COFs) often leads to insoluble powders or fragile films on solution surfaces. Here, the authors report large-area two-dimensional (2D) COF films with controllable thicknesses via vapour induced conversion.

## Introduction

Covalent organic frameworks (COFs), a class of crystalline porous polymers, have gained rapid recognition of the researchers due to their well-defined porosities, interconnected structures, and good chemical stability^1–4^. Based on these advantages, COFs enable the implementation of chemical and structural control for promising applications in the field of gas storage^5–7^, proton conduction^8,9^, and sensing^10,11^. In particular, two-dimensionally linked π-conjugated COFs (2D-COF) having an adjustable electronic band gap are a new generation of semiconducting materials for electronics^12–14^. The structures of 2D-COFs, which are chemically bonded by boronate, boroxine, hydrazone, and imine linkages, contain well-defined framework^1,3,15^. The light elements (N, O, S) can be embedded in the structures via quantitative post-modification or they can be introduced into the skeletons via the bottom-up synthesis. Furthermore, synthetic 2D organic frameworks composed of N atoms exhibit outstanding properties and functions, and have emerged as a highly tunable alternative to metal catalysts for oxygen reduction reaction (ORR)^16–18^.

In recent years, a variety of methods including solvothermal methods, ultrasonic methods, mechanical grinding and on-surface synthesis techniques have been developed for the synthesis of COFs via all of the common linkages (including imine) targeting specific practical applications^3,19–22^. 2D COF-5 particles with grain sizes of micrometers were yielded via seeded growth in solution^20^. However, it is difficult to disperse these crystalline particles fully in solution for uniform films via traditional film processing methods, e.g. spin coating and drop-coating, because of strong interactions between COF layers. Alternatively, on-surface engineering was shown to be an effective approach to 2D COF fabrication^23^. Single-crystal metals and graphite have also been demonstrated to be able to grow COF grains up to tens of nanometers in ultra-high vacuum^24–27^, and the selective growth of imine-linked COF-366 films on hexagonal boron nitride facilitates the fabrication of COF-based field-effect transistor device (FET) with high current modulation^28^. Interfacial synthesis of soluble organic building blocks into ultrathin films on solution surface offers competitive advantages in scalability and processability^29,30^, and has been applied for the preparation of uniform large-area 2D imine-linked COF films on solution surface^31,32^. Langmuir–Blodgett (LB) techniques have been used to transfer them to a substrate for film applications^33^. However, to avoid the possible damage arising from weak intermolecular forces in the film, a slow-rising process during the separation of a COF film from the liquid surface necessitates special equipment and skillful operation^30,32^, which leads to the increased production cost.

Recently, chemical vapor deposition (CVD) method was shown to be an elegant approach to 2D thin film materials through the reaction between organic or inorganic films to be deposited and the other gases to produce nonvolatic solid thin films on substrates^34–37^. Separating the polymerization process from the step at which film formation occurs, as is the case for zeolitic imidazolate framework thin films^37^, usually enables a higher-quality metal–organic frameworks (MOFs) with controllable thickness that can be directly used for the fabrication of electronic devices for microelectronics. Here we prepared a precursor–4,4′,4″,4″′-(1,3,6,8-Tetrakis(4-aminophenyl)pyrene (PyTTA)–film on growth substrate, from which a 2D COF can be constructed by Schiff base coupling of the monomers with volatile terephthalaldehyde (TPA) in a CVD system. This process is much more feasible for the realization of regular growth patterns of COFs with controllable thickness. The carrier mobility of a 30-nm-thick PyTTA-TPA COF films can reach 1.89 × 10^−3^ cm^2^V^−1^ s^−1^. Furthermore, the presence of a well-defined framework with a large pore size of ~2.4 nm in the 2D PyTTA-TPA COF film functioned as an active metal-free catalyst, facilitating ion transport and efficiently catalyzing hydrogen generation from water. The method can also be used to synthesize other 2D COF films based on PyTTA, BPyDCA ([2,2′-Bipyridine]-5,5′-dicarboxaldehyde) and BPDA (4,4′-Biphenyldicarboxaldehyde). One of the major benefits of the method is that it can avoid the need for either a particle processing process or a complicated and skilled transfer process due to the use of an atmosphere of water/acetic acid to adjust the reversible reaction directly on substrates.

## Results and discussion

### Synthesis of COF films

**Fig. 1:**
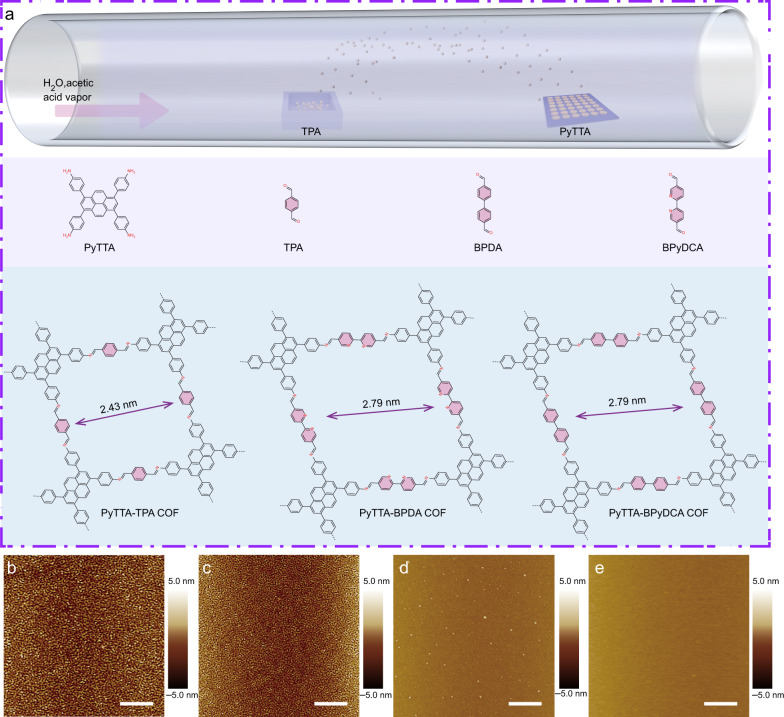
Growth and structure. **a** Schematic representation for the growth of imine-linked 2D COF films on SiO_2_/Si substrates. **b** AFM image of a 7-nm-thick PyTTA film prepared by thermal evaporation. The average roughness (*R*_*a*_) is 1.96 nm. **c**–**e** AFM images of PyTTA-TPA COF films grown at different growth times. **c** 7 h, *R*_*a*_ = 1.53 nm. **d** 14 h, *R*_*a*_ = 0.97 nm, **e** 15 h, *R*_*a*_ = 0.73 nm, Scale bar 2 μm.

PyTTA-TPA, PyTTA-BPyDCA, and PyTTA-BPDA COF films (Fig. 1a) were grown in an ambient pressure CVD system (see Methods section). The growth conditions avoid the use of specific reaction equipment, which will be useful for the large-scale production of COF film at a lower cost. To realize the controllable reaction between PyTTA and TPA, PyTTA monomer was firstly loaded on a clean growth substrate uniformly by thermal evaporation (Supplementary Figs. 1 and 2). The evaporation rate of PyTTA was maintained at 0.1 Å/s and the thickness could be precisely controlled (Supplementary Fig. 3). The growth substrate with PyTTA on its surface was then placed in a quartz tube mounted inside a tube furnace. TPA powders were placed in a ceramic boat at the upper stream side and maintained at 80 °C during the reaction. The tube furnace was externally connected with a bubbler for holding acetic acid solvent, and a hydrogen and argon flow (or pure hydrogen or argon, supplementary Figs. 4–6) was used as carrier gas to transport H_2_O, acetic acid, and TPA vapor to the PyTTA surface for COF synthesis. In the temperature range from 80 °C to 150 °C, the higher the temperature used, the faster the reaction (Supplementary Fig. 7). After careful screening, we used a growth temperature of 140 °C to obtain large-area COF films on substrates under normal pressure (Supplementary Fig. 8).

2D periodic molecular framework occurs through the formation of the imine bridging units from the condensation reaction between –CHO and –NH_2_. However, the synthesis of COF films based vapor-induced film conversion is significantly more complicated than the process in solution or at air/liquid interface. Organic monomers are in the form of single molecules in solution that can adjust their direction and angle freely during the construction of crystalline COFs, but interaction forces between the neighboring molecules in PyTTA films would inevitably limit the reactivity between the –NH_2_ and –CHO functional groups. In the presence of only TPA and PyTTA, annealing of a composite membrane did not result in crystalline PyTTA-TPA COFs (Supplementary Fig. 9). However, small molecules can intercalate into the molecule space of organic film, and weak the van der Waals interaction between neighboring monomers^37^. Figure 1b–e showed the topography change of PyTTA surface. The intrinsic PyTTA film prepared by thermal evaporation is characterized by a closely packed powder (Fig. 1b). In the presence of acetic acid and H_2_O, its surface undergoes significant modifications with continuous flattening after growth (Fig. 1c–e). Molecular dynamics (MD) simulations suggest that H_2_O molecules infiltrate the PyTTA network, increasing the space between the monomers and facilitating the condensation reaction between PyTTA and TPA (Supplementary Fig. 10, simulation details were provided in SI)^38^.

### Characterizations of PyTTA-TPA COF films

**Fig. 2:**
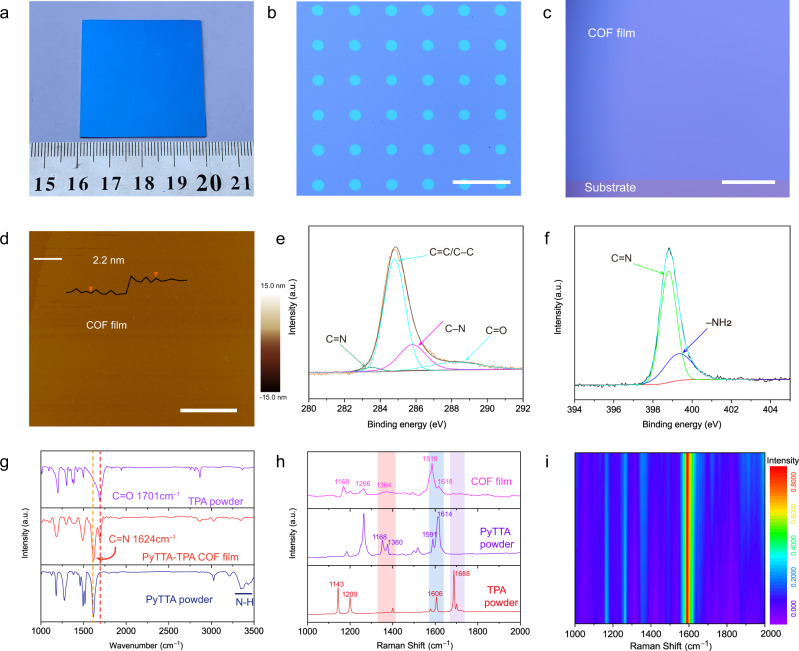
Morphology and spectral characterizations of PyTTA-TPA COF films. **a** Photograph of 2D PyTTA-TPA COF film grown on a SiO_2_/Si substrate. **b** Optical image of PyTTA-TPA COF patterns. Scale bar 100 μm. **c** High-magnification optical image of the PyTTA-TPA COF film. Scale bar 50 μm. **d** AFM image of the 2D PyTTA-TPA COF film with a thickness of ~2.2 nm. Scale bar 2 μm. **e**, **f** C1s and N1s XPS spectra of 30-nm-thick PyTTA-TPA COF film. **g** FT-IR spectra of 30-nm-thick PyTTA-TPA COF film, PyTTA and TPA powder. **h** Raman spectra of 30-nm-thick PyTTA-TPA COF film, PyTTA and TPA COF powder. **i** A contour map with multiple sets of normalized Raman data selected from Raman mapping data.

Various substrates such as SiO_2_/Si, glass, Cu foil, and so on have been used to grow PyTTA-TPA COF films (Fig. 2a and Supplementary Figs. 11–16). The thickness of the films can be easily controlled from several nanometers to tens of nanometers by adjusting the pre-arranged PyTTA films (Supplementary Figs. 3, 17, and 18). Because the growth occurs on substrate surface at atmospheric pressure, it follows that patterning PyTTA film through a shadow mask will promote the growth of COF patterns (Fig. 2b and Supplementary Fig. 19), which is compatible with current silicon processing techniques and highly desired for 2D electronics. Figure 2c shows a high-magnification optical microscopy image of the COF film. The uniform color contrast indicates the film is of uniform thickness, as is also confirmed by scanning electron microscope (SEM) observation (Supplementary Fig. 20). Atomic force microscopy (AFM) was used to measure the detailed feature of the PyTTA-TPA COF film (Fig. 2d), which indicated a smooth surface (Ra = 0.731 nm) with a step height of 2.2 nm, corresponding to few-layer 2D COFs.

XPS survey spectrum of the PyTTA-TPA COF film shows the presence of carbon and nitrogen along with the signals of Si and O from the SiO_2_/Si substrate (Supplementary Fig. 21). The C1*s* XPS spectrum can be divided into several major peaks centered at around 283.5 eV, 284.8 eV, 285.8 eV, and 288.2 eV (Fig. 2e), which correspond to the C=N, C–C/C=C, C–N, and C=N, respectively, with a graphite-like structure^39^. Similarly, the high-resolution XPS spectrum of N 1 *s* is composed of two peaks which are centered at around 399.4 and 398.9 eV (Fig. 2f), and can be assigned to the N of the unreacted amine groups in PyTTA and the N in imine bonding structure, respectively^39^. According to the FT-IR spectra (Fig. 2g), adsorption bands of the −NH_2_ groups of PyTTA located at 3334 and 3231 cm^−1^ disappear after growth with the subsequent appearance of a peak at 1624 cm^−1^, corresponding to the C=N stretching modes of imines, indicating the formation of –C=N– linkages in the PyTTA-TPA COFs (Fig. 2g)^39^. Based on the change of C=N: –NH_2_ peak intensity, it is easy to understand that the condensation reaction between amines and aldehydes is enhanced with the extension of reaction time (Supplementary Figs. 22–24)^40,41^.

For further analysis of the structure and uniformity of the COF films, Raman spectra were measured using a confocal Raman microscope. Compared with those of PyTTA and TPA precursors, a broad band from 1660 cm^−1^ to 1720 cm^−1^ corresponds to the aldehyde C = O stretching vibration, while the band at 1364 cm^−1^ is derived from the stretching vibration of –NH_2_ (Fig. 2h)^39^. The appearance of strong peaks at 1591 cm^−1^ and 1618 cm^−1^ correspond to the stretching vibrations of the aromatic C=C groups and the imine moieties, respectively, indicating the formation of the imine bond linking unit^39^. Spatial dependences of the intensity of the characteristic Raman peak at 1591 cm^−1^ were plotted in Fig. 2i and Supplementary Fig. 25. The uniform color intensity is a reliable indicator of the uniform thickness. The UV-Vis spectrum of PyTTA-TPA COF film shows a broad absorbance mainly in the range from 250 and 600 nm, which is designated as the π-π transition (Supplementary Fig. 26). The energy band gap is ~1.87 eV, calculated from the UV-Vis edge absorption (Supplementary Fig. 27), and the lowest unoccupied molecular orbital (LUMO) measured by cyclic voltammetry (CV) is –3.73 eV (Supplementary Fig. S28). Combining the energy band gap and LUMO level, the highest occupied molecular orbital (HOMO) energy level calculated is around –5.60 eV, suggesting the semiconducting character of the PyTTA-TPA COF film (Supplementary Fig. 29).

**Fig. 3:**
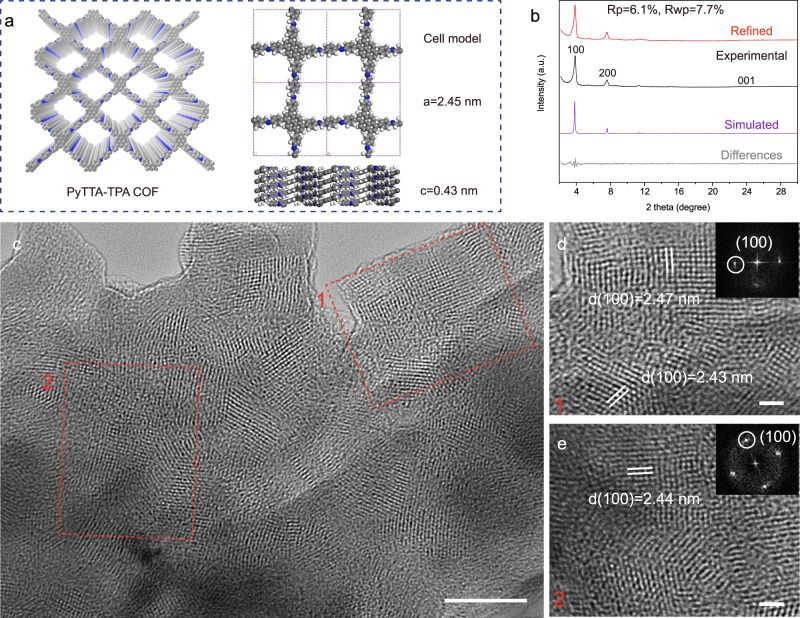
GIWAXS and TEM characterizations of PyTTA-TPA COF films. **a** Simulated molecular structure of PyTTA-TPA COF based on AA-stacking mode. **b** Theoretical and experimental GIWAXS of the PyTTA-TPA COF film with a thickness of ~30 nm. **c** TEM of the PyTTA-TPA COF film with a thickness of ~13 nm. Scale bar 50 nm. **d**, **e** TEM of the PyTTA-TPA COF film captured from regions in **c**. Scale bar 10 nm. The inset showed the fast Fourier transformation (FFT) patterns.

Grazing-incidence wide-angle X-ray scattering (GIWAXS) was used for characterization of the crystal structure of PyTTA-TPA COF films, and the crystallographic information of PyTTA-TPA COF film was obtained by Pawley refinement of film diffraction signal (Fig. 3a, b). According to the Pawley refinement results (Fig. 3b and Supplementary Table 1), the PyTTA-TPA COF belonged to the C2/m space group with unit cell parameter of *a* = 2. 5 nm, *b* = 2.4 nm, and *c* = 0.4 nm. *α* = 90°, *β* = 91°, and *γ* = 90°. Three prominent diffraction peaks at 2θ = ~3.7°, ~7.5° and ~23.4° can be assigned to the (100), (200) and (001) facets, respectively^42^. The presence of a high intensity peak at 3.7° is associated with the periodicities in (100) plane and is indicative of a well-defined ordered columnar array. The crystal size of the PyTTA-TPA COFs is ~26.8 nm by Scherrer’s analysis, which is comparable to that of PyTTA-TPA COF powders (~22.0 nm) synthesized by solvothermal method (Supplementary Fig. 30). The 2D GIWAXS in Supplementary Fig. 12 showed an arc pattern, and no orientation was found in the COF film, which indicated the crystallization process of irregular polygons to layered frameworks with ordered pores was driven by *π*–*π* stacking interactions of adjacent sheets^41^. However, on highly oriented pyrolytic graphite (HOPG) (Supplementary Fig. 31), the drastic change in intensity of the (001) signal in the directions proves the formation of oriented COF crystalline regions that preferably lie with the *ab* plane parallel to the HOPG surface^43^. The results indicated that PyTTA and TPA precursors can move/rotate on substrates for COF assembly at the aid of H_2_O (Supplementary Fig. 9). Transmission electron microscopy (TEM) provides the most direct evidence of the microstructure of the COF film with the grain size reaching 50 nm (Fig. 3c). High-resolution TEM (HRTEM) (Fig. 3d, e) demonstrates a clear quadrilateral network structure with a pore size of ~2.45 nm (Fig. 1a), which is also consistent with the calculated pore size of PyTTA-TPA COFs by GIWAXS (Fig. 3b).

**Fig. 4:**
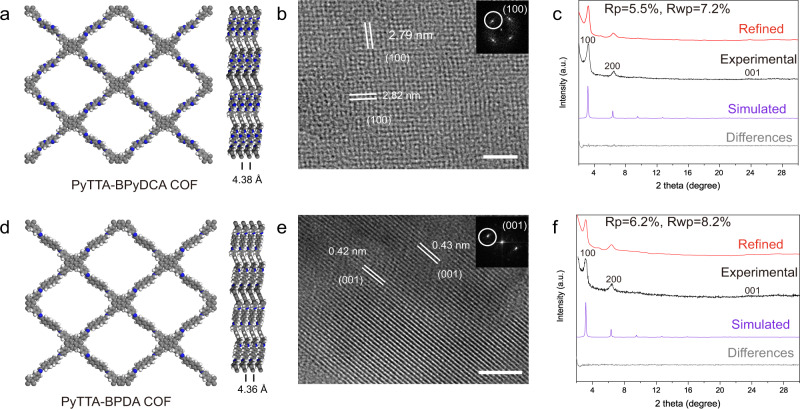
GIWAXS and TEM characterizations of PyTTA-BPyDCA and PyTTA-BPDA COF films. **a** Simulated molecular structure of PyTTA-BPyDCA COF based on AA-stacking mode. **b** HRTEM of the PyTTA-BPyDCA COF film. Scale bar 20 nm. **c** Theoretical and experimental GIWAXS of the PyTTA-BPyDCA film. **d** Simulated molecular structure of PyTTA-BPDA COF based on AA-stacking mode. **e** HRTEM of the PyTTA-BPDA COF film. Scale bar 5 nm. **f** Theoretical and experimental GIWAXS of the PyTTA-BPDA film. The thickness of PyTTA**-**BPyDCA and PyTTA-BPDA COF films is about 41 nm and 37 nm respectively.

To check the generality of the CVD-based approach for growing 2D COF films, the synthesis of two other COFs based on PyTTA-BPyDCA and PyTTA-BPDA were studied (Fig. 4 and Supplementary Fig. 32). Except for the use of BPyDCA and BPDA instead of TPA as precursors, the growth procedure and parameters were similar to those of PyTTA-TPA COF films. Raman, XPS, and FTIR measurements verified the formation of imine bonds (Supplementary Figs. 33–35). As compared to the PyTTA-TPA COF, lattice constant of ~2.8 nm was observed for PyTTA-BPyDCA COF films (Figs. 4b and 1a)^44^, which is consistent with the crystal plane along the (001) direction of the theoretical simulation structure. The two-dimensional slices with an interplanar spacing of ~0.43 nm indicate a highly ordered arrangement in the COF film (Fig. 4e). GIWAX data of PyTTA-BPyDCA and PyTTA-BPDA films matches the theoretical calculation (Fig. 4c, f and Supplementary Tables 2 and 3). The peaks located at ~3.2°, ~6.4° are assigned to the (100) and (200) facets, respectively^44^.

### Electrical performance

**Fig. 5:**
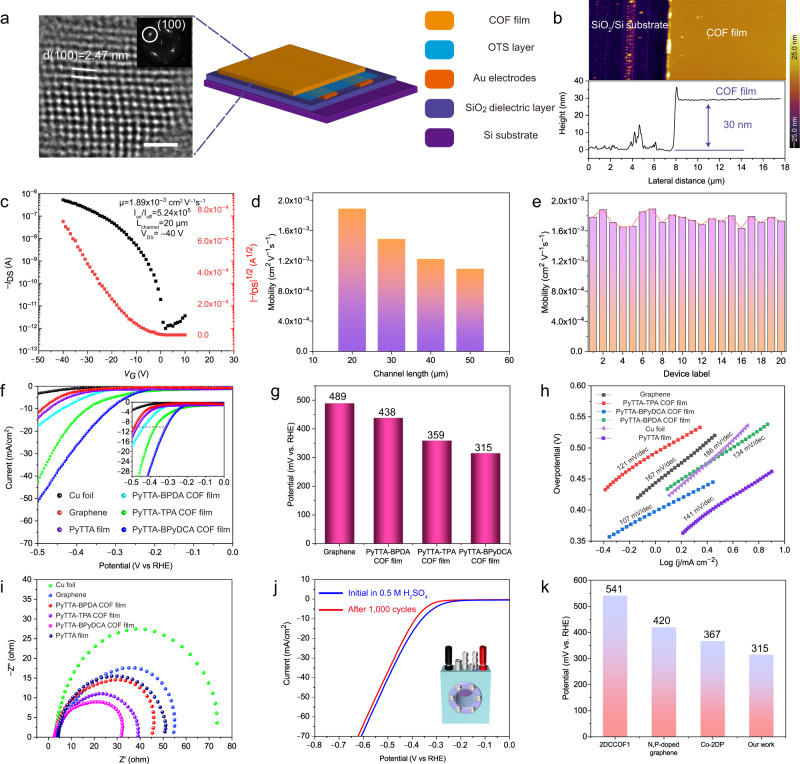
Electrical properties of COF films. **a** Schematic diagram of a FET device using PyTTA-TPA film as working materials. Scale bar 10 nm. **b** AFM image of the PyTTA-TPA COF film used for FETs with a thickness of ~30 nm. Scale bar 4 μm. **c** Transfer characteristics (*I*_DS_ vs *V*_G_) of the PyTTA-TPA COF device at *V*_DS_ = −40 V. **d** Hole mobility of the PyTTA-TPA COF-based FET devices under different channel lengths. **e** Distribution histogram of hole mobility. **f** LSV curves of Cu foil, graphene, PyTTA-TPA, PyTTA-BPyDCA, and PyTTA-BPDA COF films in 0.5 M H_2_SO_4_. **g** Overpotential of graphene, PyTTA-TPA, PyTTA-BPyDCA, and PyTTA-BPDA COF films at current density of 10 mA cm^−2^. **h** Tafel plots of Cu foil, graphene, PyTTA-TPA, PyTTA-BPyDCA, and PyTTA-BPDA COF films. **i** Nyquist plots of Cu foil, graphene, PyTTA-TPA, PyTTA-BPyDCA, and PyTTA-BPDA COF films. **j** LSV plots of PyTTA-TPA COF film before and after 1000 CV cycles. **k** Compared with reported materials^54,55,57^.

Imine-linked COFs, a class of π-conjugated semiconducting polymer, have a porous, well-defined network with large surface area, which is suitable for the applications in electronics and catalyst^14^. To study the electronic properties of the COF films in field-effect transistors (FETs), 30 nm PyTTA-TPA COF films were grown in situ on a OTS-modified SiO_2_/Si substrate^45–47^, which act as the active layer, while gold was used as the source and drain electrode for the device (Fig. 5a, b, Supplementary Figs. 36–38). Self-assemble OTS monomers have proved to be excellent candidates for ultra-thin gate dielectric layers in organic field-effect transistors (OFETs)^48–51^. In the *I*_DS_–*V*_DS_ characteristic curve, the device exhibited a saturation current of ~9.0 × 10^−7^ A at a gate voltage of –40 V, and the threshold voltage is <–5 V (Supplementary Fig. 39a). In Fig. 5c, the *I*_DS_–*V*_G_ characteristics for the device in N_2_ show a rapid increase of the source-drain current (*I*_DS_) with the increase of negative gate voltage (*V*_G_). The transistor exhibits a *p*-channel behavior with a hole mobility of ~1.89 × 10^−3^ cm^2^ V^−1^ s^−1^, estimated from the saturation current, and a high on/off ratio of 10^5^. The mobility is three orders of magnitude higher than that of previously reported imine-linked 2D conjugated COF film because the direct growth of COF films on SiO_2_/Si substrates avoided the damage of films caused by transfer^12,44^. Notably, the value is also significantly higher than that of PyTTA precursors (Supplementary Fig. 40), indicating the transport is due to COF lattice, not just hoping between unreacted precursor molecules. According to four-probe measurements, the conductivity of the film is ~8.40 × 10^−6^ S/cm (Supplementary Fig. 41). Generally, grain boundaries in semiconductor thin films will trap carriers, resulting in a decrease in the number of movable carriers. Therefore, an appropriate reduction in channel length will help improve device performance (Fig. 5d and Supplementary Fig. 42). Additionally, we have drawn a histogram based on the carrier mobility data of 20 FET devices (Fig. 5e), confirming the uniformity of the COF film quality.

To further explore the potential applications of the porous 2D COF films, the electrocatalytic activities of PyTTA-TPA, PyTTA-BPyDCA, and PyTTA-BPDA COF films directly grown on copper electrodes were evaluated towards HER in acidic (0.5 M H_2_SO_4_) aqueous solution (Fig. 5f). The results of the linear sweep volt ampere curves indicated negligible electrocatalytic activity of the bare copper substrate, as reflected by the higher overpotential and lower current density^52–54^, while better catalytic activity was shown by the three COF films. In presence of –10 mA cm^−2^ as the driving current density, the respective HER onset potentials of PyTTA-TPA, PyTTA-BPyDCA, and PyTTA-BPDA COF films were 235, 208, and 268 mV while the overpotential values were 359, 315, and 438 mV, respectively (Fig. 5f). However, the electrocatalytic activity of pure graphene and PyTTA precursors is negligible, indicating that the COF structure is mainly responsible for the observed electrocatalytic effect. These results are better than those of metal-free COFs^30,55^, and compare favorably with those obtained using some traditional metallic catalysts^56^, indicating extraordinary activity toward HER. In our experiments, we observed an increasing trend of the onset potential from PyTTA-BPyDCA to PyTTA-TPA and to PyTTA-BPDA COF (Fig. 5g). DFT calculations were performed to investigate the origin of HER performance of these COFs. Our results showed that the free energy changes for the HER of various N sites of these COFs at equilibrium potential (0 V) are comparable with each other and are all close to 0 eV (Supplementary Fig. 43, calculation details were provided in SI), suggesting the high HER performance of these COFs. Moreover, the small free energy changes of these COFs also suggest that increasing proportion of the N heteroatom will improve HER catalytic activity, as consistent with our experiments. As shown in Fig. 5h, the Tafel slopes of these COFs varied from 134 to 107 mV/decade. The results indicate that the rate-determining step of the whole HER process was adsorption of the initial proton (i.e. the Volmer reaction, H^+^ + e^−^ = H*, where H* refers to the chemically adsorbed hydrogen atom on the active site in the electrocatalytic reaction), as is consistent with previous reports^57^.

Electrochemical impedance spectroscopic (EIS) measurements are studied to investigate the electrode dynamics from the change of impedance with the frequency of sine wave. Figure 5i shows the Nyquist plots of the COF film-based catalysts within the frequency range of 0.01 Hz to 100 kHz at –50 mV vs RHE. The semicircular outline indicates a kinetically controlled electrochemical reaction on the electrode surface. The calculated charge-transfer resistance (*R*_ct_) for the PyTTA-BPDA, PyTTA-TPA, and PyTTA-BPyDCA COF films was ~45.8, ~38.9, and ~31.5 Ω, respectively, which is smaller than that of some reported metal-free catalysts, suggesting rapid charge-transport in the imine-linked COFs. To investigate the durability of the COF film, we conducted a long-term potential cycling test under the potential range of 0 V to –0.8 V vs RHE at a scan rate of 1 mV s^−1^. After 1000 cycles, the activity of the PyTTA-TPA film remained changed with respect to the current density at the onset potential or different potentials (Fig. 5j), suggesting high stability of the PyTTA-TPA film. The high HER catalytic activity may originate from the perfect contact to Cu electrode. Due to the absence of adhesives, contact resistance will be reduced by creating a clean interface, and thus promoting the electronic transmission between the catalyst and electrode^50,55,57^. The vapor-induced approach provides uniform and stable COF films grown on electrodes, leading to a highly adjustable catalytic platform for HER (Fig. 5k).

## Discussion

In summary, we demonstrated a vapor-induced process for synthesizing high-quality 2D PyTTA-TPA, PyTTA-BPyDCA, and PyTTA-BPDA COF films on a large scale. Despite the topological limitations from the perspective of close-packed molecular arrangement, the thermodynamic control of the crystal structure is increased due to increasing reversibility of the Schiff base reaction in the presence of acetic acid and H_2_O. These COF films can be grown on various substrates and can be directly used for device fabrication, thus avoiding the problems of film contamination and breakage associated with the preparation of films by traditional solution-based methods. TEM, AFM, and GIWAXS measurements illustrated high quality of these COF films with the carrier mobility up to 1.89 × 10^−3^ cm^2^ V^−1^ s^−1^ and a high on/off ratio of 10^5^. When used as active materials for HER, the COF films show low onset potentials and overpotentials with high stability. This work contributes a general vapor-induced method for producing large-area 2D COF films with direct relevance to FETs and HER. Our work further points to the possibilities of using the method for the growth of other 2D COFs, which is important for basic research and practical applications.

## Methods

### Preparation of PyTTA film

PyTTA film was deposited on SiO_2_/Si, glass, Cu foil and so on via thermal evaporation. In all, 20 mg PyTTA powders were placed into a crucible in an inert gas organic evaporation system, and growth substrates were loaded with their face down to the PyTTA source. PyTTA can be patterned on substrates by using a shadow mask. When the vacuum reaches 8 × 10^−6^ mbar, the temperature rises to 130 °C at a rate of 20 °C/min, and then slowly rises to 180 °C at a rate of 5 °C/min. The evaporation rate was maintained at 0.1 Å/s, and the thickness of PyTTA film can be controlled by deposition time.

### Synthesis of 2D COF films

PyTTA-TPA, PyTTA-BPyDCA, and PyTTA-BPDA COF films were grown on various substrates by vapor-induced conversion in a CVD system. The growth substrates with PyTTA films on their surface were placed in the middle of the 2-inch quartz tube, which was installed in the tube furnace. TPA (or BPyDCA, BPDA) powders were placed in a separate ceramic boat at the upper stream side. The tube furnace is externally connected with a bubbler to hold a 20 ml deionized aqueous solution of acetic acid (Vacid/Vwater = 9:1) and a hydrogen and argon flow of 10 sccm and 10 sccm is used as carrier gas. The center heating zone was heated to 140 °C. Note that the temperature of the TPA (or BPyDCA, BPDA) powders was controlled at ∼80 °C (105 °C for BPyDCA, 110 °C for BPDA) when the center heating zone reaches 140 °C. After a 7-day growth, the central heating zone was heated to 180 °C for 1 h to remove unreacted TPA (or BPyDCA, BPDA) precursors. The furnace was then cooled to room temperature to obtain COF films.

### Characterizations of 2D COF films

Film morphology was imaged using a NIKON Eclipse LV-100 optical microscopy. The material structure of COF films was studied by using a Horiba Raman spectroscope with laser excitation at 532 nm at room temperature. SEM images were obtained by a Hitachi S-4800 scanning electron microscope. Transmission electron microscope (TEM, JEM-2100F) was operated at a voltage of 200 kV. The crystal structure was measured by using 2D grazing-incidence wide-angle X-ray scattering (2D-GIWAXS, XEUSS). Grazing incidence WAXS measurements (GIWAXS) were performed on a Xeuss 2.0 SAXS/WAXS system (Xenocs SA, France). Cu Kα X-ray source (GeniX3D Cu ULD), generated at 50 kV and 0.6 mA, was utilized to produce X-ray radiation with a wavelength of 1.5418 Å. A semiconductor detector (Pilatus 300 K, DECTRIS, Swiss) with a resolution of 487 × 619 pixels (pixel size = 172 × 172 μm^2^) was used to collect the scattering signals. X-ray photoelectron spectroscopy (XPS) was conducted with an ESCALAB250Xl spectrometer using an Al Kα X-rays as the excitation source. Fourier transform infrared (FTIR) spectroscopy was carried out on RT-DLaTGS 27 spectrometer. AFM images were obtained using CyPher ES Atomic Force Microscope (AFM). Structural modeling of COFs was generated using the Materials Studio program employing the Building (Crystal) module, and the lattice parameters were geometrically optimized using the universal force-filed in the forcite module and further optimized by the DFTB + module with 3ob Slater-Koster parameterization set and a 2 × 2 × 5 K-point mesh. The force on each atom in the unit cells of COFs was optimized to be <0.5 Kcal/mol/Å and an energy convergence of 0.05 Kcal/mol was chosen. The Pawley fitting (Reflex module) was performed to fit the lattice parameters iteratively until the Rwp value converges and the overlay of the observed with refined profiles shows good agreement. The final *Rp* and *Rwp* values were ~6.1% and 7.7% (PyTTA-TPA COF film), respectively.

### Fabrication of FETs

To avoid the damage to the COF film caused by the evaporation of the source and drain electrodes, the bottom-gate bottom-contact (BG/BC) device structure was adopted to construct the COF-based field effect transistor (FET) device. SiO_2_/Si chip with 30 nm Au source–drain electrodes on its surface were purchased from Micro-Nano Fabrication Laboratory of Peking University. The thickness of the SiO_2_ dielectric layer is 300 nm. SiO_2_/Si chip was modified by OTS^45–47^. PyTTA monomer with different thickness was loaded on the OTS-modified growth substrate uniformly by thermal evaporation. Finally, the growth substrates were placed in a quartz tube mounted inside a tube furnace for COF growth. The electrical properties were measured by a Keithley 4200SC semiconductor parameter analyzer. The FET measurements were carried out in Glove box at room temperature. The carrier mobility (μ) data was calculated in the saturation region according to the following equation:

$${I}_{{{{{{{\mathrm{DS}}}}}}}}=\frac{W}{2L}{C}_{{{{{{\mathrm{i}}}}}}}{{{{{\rm{\mu }}}}}}{({V}_{{{{{{{\mathrm{GS}}}}}}}}-{V}_{{{{{{{\mathrm{th}}}}}}}})}^{2}$$

where *I*_DS_ is the source-drain current, *W* and *L* are the channel width and length, respectively, *V*_GS_ is the gate voltage, *C*_i_ is the capacitance per unit area of the dielectric layer, and *V*_th_ is the threshold voltage.

### Electrochemical measurements

Electrochemical measurements were conducted using a CHI 760E electrochemical station via the standard three-electrode electrolytic system. A COF film grown on a piece of Cu foil is used as working electrode, and an Ag/AgCl (saturated KCl) electrode and a piece of Pt foil are used as reference electrode and counter electrode respectively. The film thickness is ~13 nm. Before the electrochemical measurement, the electrolyte (0.5 M H_2_SO_4_) was purged with pure N_2_ for 30 min to eliminate dissolved oxygen. HER activities were carried out by linear sweep voltammetry (LSV) with a scan rate of 5 mV/s. The potentials were calibrated to the reversible hydrogen electrode (RHE) according to the Nernst equation (*E*_RHE_ = *E*_Ag/AgCl_ + 0.0591 pH + *E*^0^_Ag/AgCl_) and iR corrected. EIS measurements were conducted at the open-circuit voltage from 1000 kHz to 0.01 Hz with an amplitude of 10 mV.

## Supplementary information

Supplementary Information

## Data Availability

The authors declare that the experimental data supporting the results of this study can be found in the paper and its Supplementary Information file. The experimental results of the study along with other simulation data are provided in the Supplementary Information file. The detailed simulation files for the study are available from the corresponding author upon request.
