# Supplementary material for: Two-dimensional covalent organic framework films prepared on various substrates through vapor induced conversion
Source: Nat Commun. 2022 Mar 17;13:1411. doi: 10.1038/s41467-022-29050-9 (PMC8931112; doi:10.1038/s41467-022-29050-9)
Supplement: Supplementary file 1 — Supplementary Information [file 41467_2022_29050_MOESM1_ESM.pdf]

Supplementary Information

Two-dimensional covalent organic framework films prepared on various substrates through vapor induced conversion

Minghui Liu[1,2], *Youxing Liu*[1,2], *Jichen Dong*[1,2], *Yichao Bai*[1,2], *Wenqiang Gao*[1,2], *Shengcong Shang*[1,2], *Xinyu Wang*[1,2], *Junhua Kuang*[1,2], *Changsheng Du*[1,2], *Ye Zou*[1,2], *Jianyi Chen*[1,2], *Yunqi Liu*[1,2]

[1]Beijing National Laboratory for Molecular Sciences, Key Laboratory of Organic Solids, Institute of Chemistry, Chinese Academy of Sciences, Beijing 100190, P.R. China

[2]University of Chinese Academy of Sciences, Beijing 100049, P.R. China

Correspondence and requests for materials should be addressed to J.C. (email: chenjy@iccas.ac.cn) or to Y.L. (email: liuyq@iccas.ac.cn)

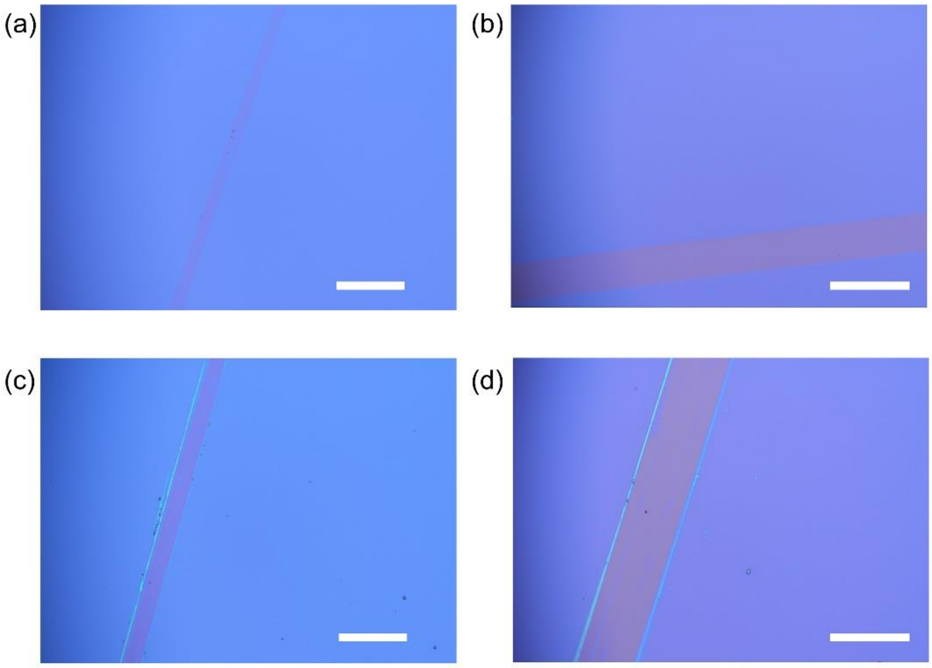

Supplementary Figure 1. OM images of PyTTA films deposited on SiO_2/Si surface with different thickness. (**a**, **b**) OM images of a 2-nm PyTTA films with different magnification. (c, d) OM images of a 4-nm PyTTA films with different magnification. Scale bar 100 μm in (a) and (c). Scale bar 50 μm in (b) and (d). The PyTTA films are uniform under optical microscope. The thickness is controlled by thermal evaporation technique.

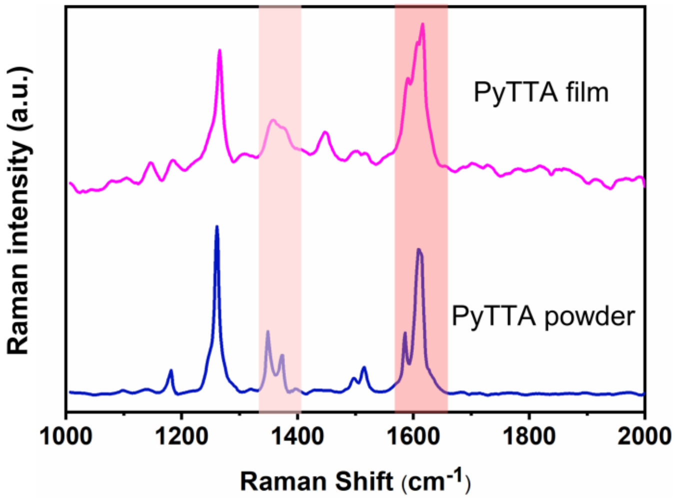

Supplementary Figure 2. Raman spectra of PyTTA powders and PyTTA film. The PyTTA film prepared via thermal evaporation shows similar Raman characteristics with those of PyTTA powders, indicating that the structure of PyTTA monomers was not damaged by thermal evaporation at 180 ℃. The thickness of PyTTA film is about 14 nm.

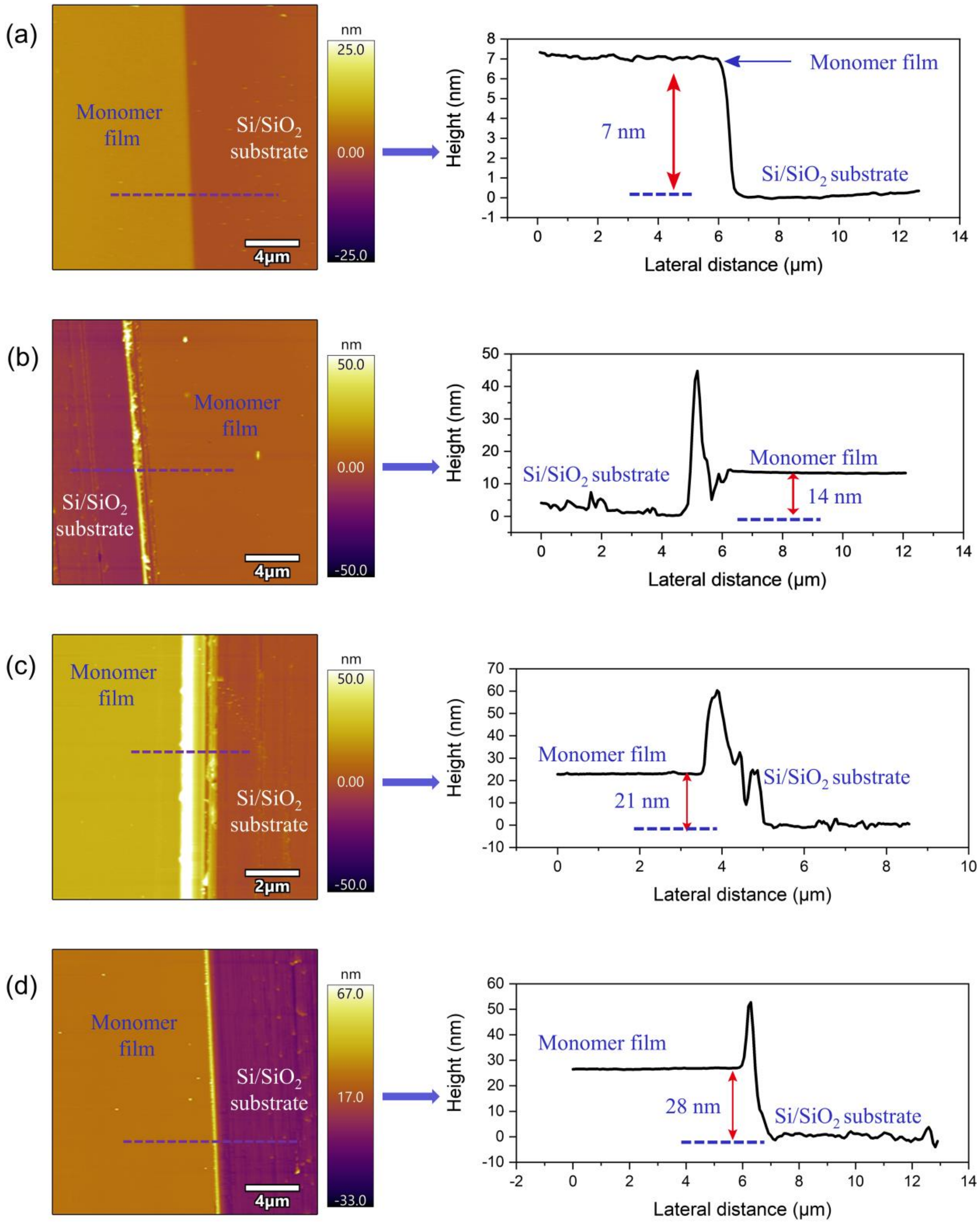

Supplementary Figure 3. AFM images of PyTTA films deposited on SiO_2/Si substrates via thermal evaporation. (a) 7 nm. (b) 14 nm. (c) 21 nm. (d) 28 nm. The thickness can be controlled by deposition time. Evaporation temperature was 180 ℃, and the evaporation rate was maintained at 0.1 Å/s.

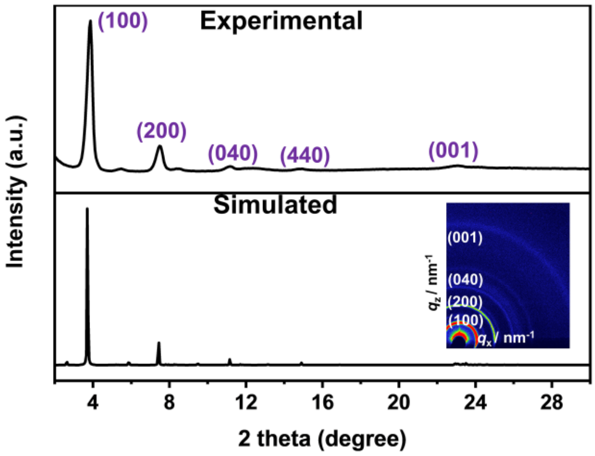

Supplementary Figure 4. GIWAXS and simulated XRD of PyTTA-TPA COF film grown on SiO_2/Si substrate. 20 sccm H_2 is used as carrier gas.

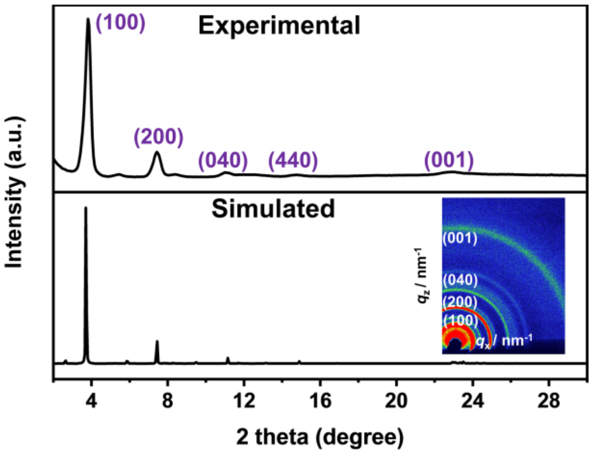

Supplementary Figure 5. GIWAXS and simulated XRD of PyTTA-TPA COF film grown on SiO_2/Si substrate. 20 sccm Ar is used as carrier gas.

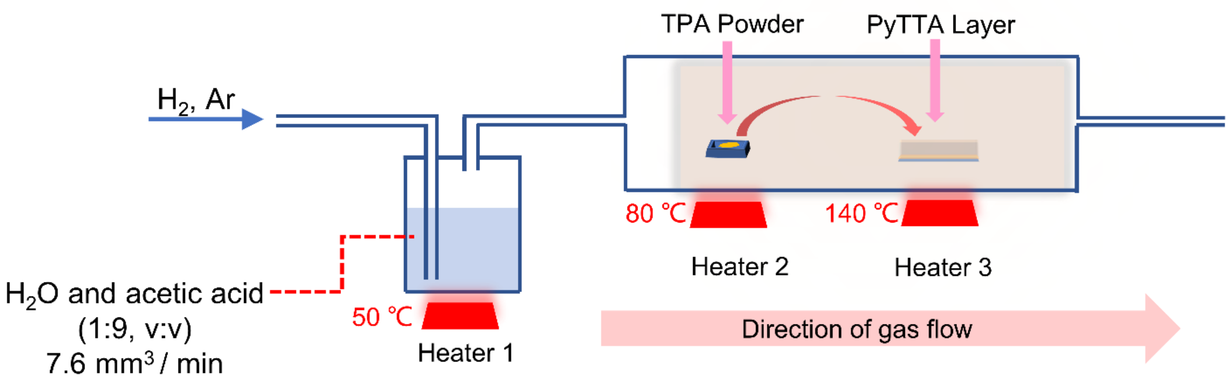

Supplementary Figure 6. Schematic diagram of the vapor induced growth of COF films in a CVD system.

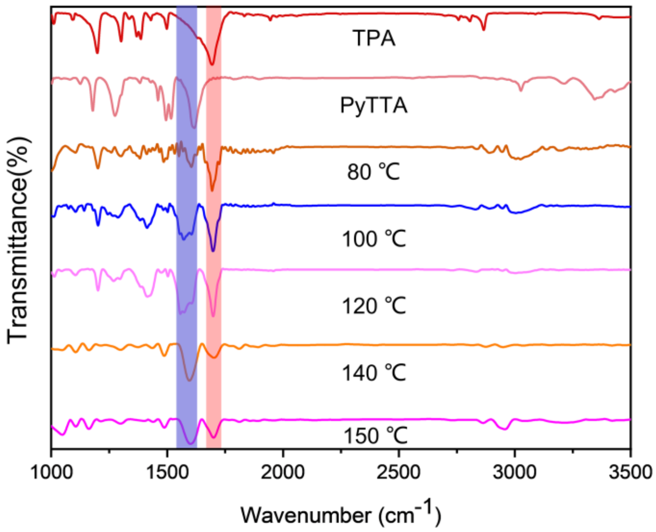

Supplementary Figure 7. FT-IR spectra of PyTTA-TPA COF films grown at different temperatures. The growth time is 14 h. The thickness of PyTTA film using for the growth of PyTTA-TPA COF film is ~ 14 nm.

With the increase of the reaction temperature, the intensity of the newly appeared –C=N– (~1624 cm^{-1}) peak increased, and the corresponding –C=O (~1701 cm^{-1}) and $–NH_2$ (~3200–3500 cm^{-1}) peak intensity decreased. The results indicate that a high reaction temperature can speed up the Schiff-base reaction.

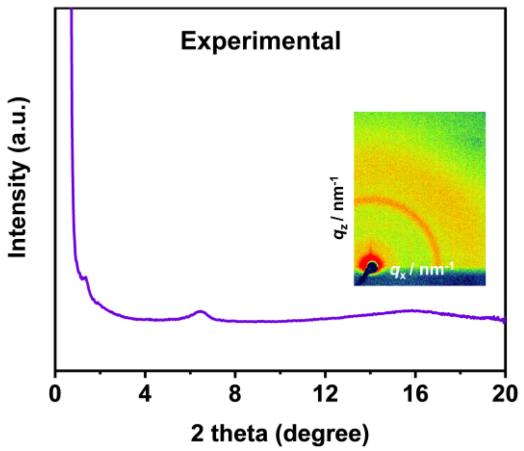

Supplementary Figure 8. GIWAXS of PyTTA-TPA COF film grown under low pressure. The growth substrates with PyTTA films on their surface were placed in a quartz tube mounted inside a tube furnace. TPA powders were placed in a separate ceramic boat at the upper stream side maintained at 80 ℃ during the reaction. CVD system was connected to a bottle of acetic acid aqueous solution. Acetic acid and H_2O gas were pumped into the CVD system and grown for 7 days at low pressure (~7 x 10^{-2} Pa). There is almost no signal for PyTTA-TPA COF (2θ = ~3.7 °, ~7.5 ° and ~23.4 °) because the concentration of TPA is very low in the CVD system. Therefore, COF film was grown under normal pressure if not specified.

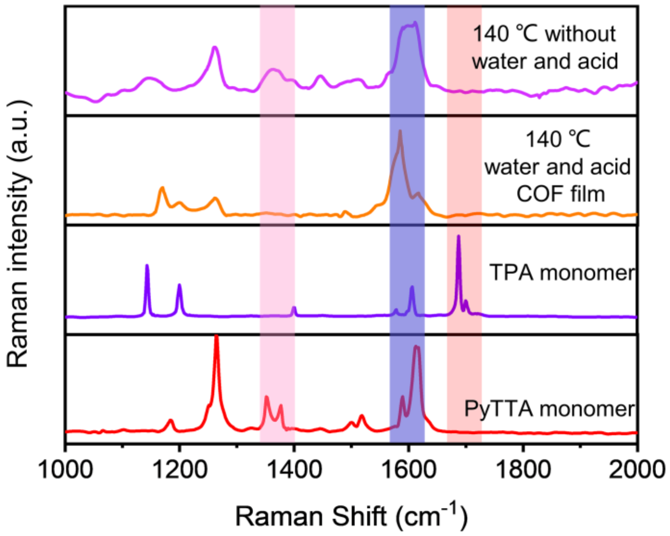

Supplementary Figure 9. Raman spectra of PyTTA, TPA and PyTTA-TPA films.

Raman was carried out to study the effects of water and acetic acid on the growth of COF films. Compared with the results of the spectrum without water and acetic acid (Purple line), the Raman peak at 1364 cm^{-1} (corresponding to the vibration mode of free amino groups) of the COF film (Orange line) was significantly weakened, and the characteristic peaks of the aldehyde group vibration corresponding to 1688 cm^{-1} and 1710 cm^{-1} also disappeared, indicating that the two precursors were effectively reaction, The results indicated that the presence of water and acetic acid is essential for the growth of COF films[1–4].

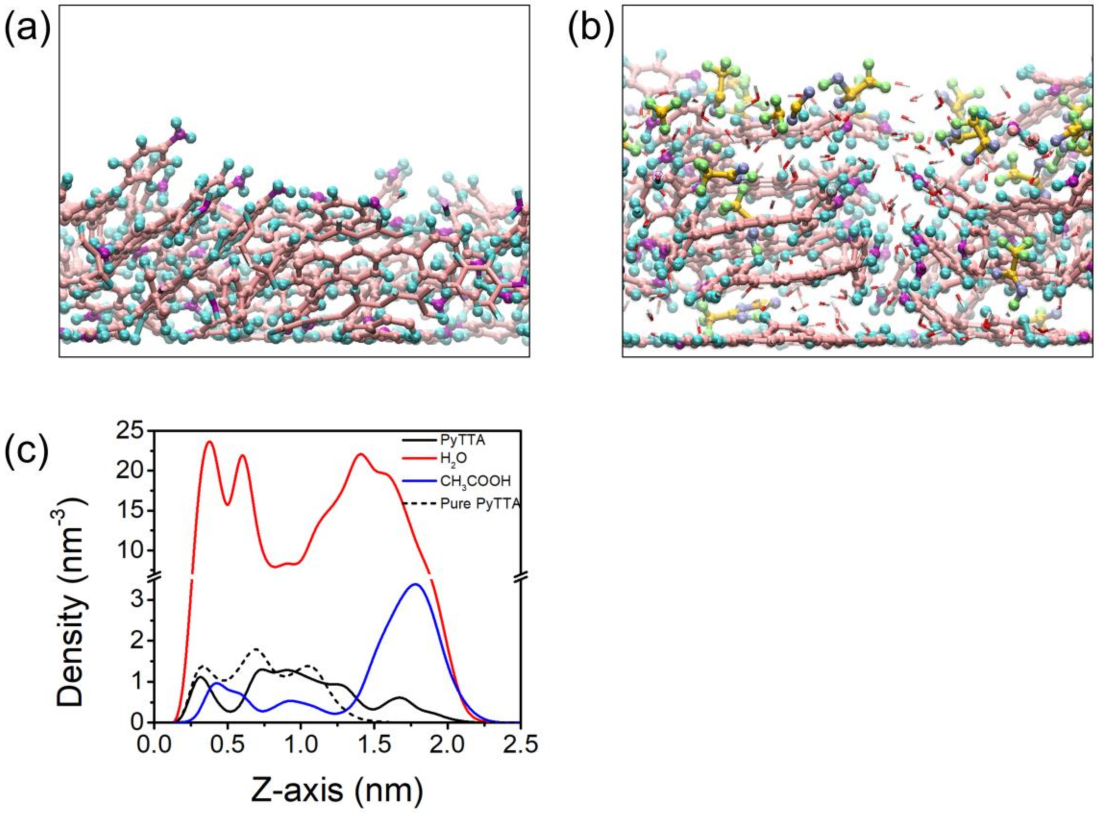

Supplementary Figure 10. Structural changes of the PyTTA precursor film calculated by DFT. (a) Schematic diagram of the PyTTA precursor film. (b) Schematic diagram of the PyTTA precursor film in the presence of water and acetic acid vapor. C, N and H atoms in PyTTA are represented by pink, purple and cyan spheres. C, O and H atoms in CH_3COOH are represented by yellow, ice blue and green spheres. H_2O molecules are represented by thin lines. (c) Spatial distribution of molecules in the z-axis direction in the presence of water and acetic acid vapor. Compared with that of pure PyTTA monomers (Dotted line), the space (Z-axis) of PyTTA monomers increased with the introduction of H_2O and CH_3COOH into a PyTTA system (Black line).

Molecular dynamics simulations were performed via the Large-scale Atomic/Molecular Massively Parallel Simulator (LAMMPS) (LAMMPS - A flexible simulation tool for particle-based materials modeling at the atomic, meso, and continuum scales)[5]. The atomic interactions are described by the ReaxFF reactive force field with low gradient corrections accounting for weak vdW interactions, which has been successfully applied in investigations of organic systems[6]. The movement of atoms was updated through the Velocity Verlet algorithm with a time step of 0.25 fs[7]. The NVT ensemble was employed through the Nose-Hoover thermostat with the temperature kept at

300 K[8,9]. Two systems with unit cells containing 12 PyTTA molecules and 12 PyTTA + 204 H_2O + 20 CH_3COOH molecules were constructed to demonstrate the effect of H_2O on the COF polymerization process. The unit cell size was set to be 30 × 30 × 60 $Å^3$ with the periodic boundary condition along X and Y axes. To mimic the effect of substrate, a 12-6 Lennard-Jones type van der Waals wall was added at the bottom of the unit cell, and the depth of the potential wall is 0.043 eV at an equilibrium distance at 2 Å. During simulation, 10 million MD steps were performed for each system, and the density distribution profiles were obtained by averaging the last 5 million MD steps.

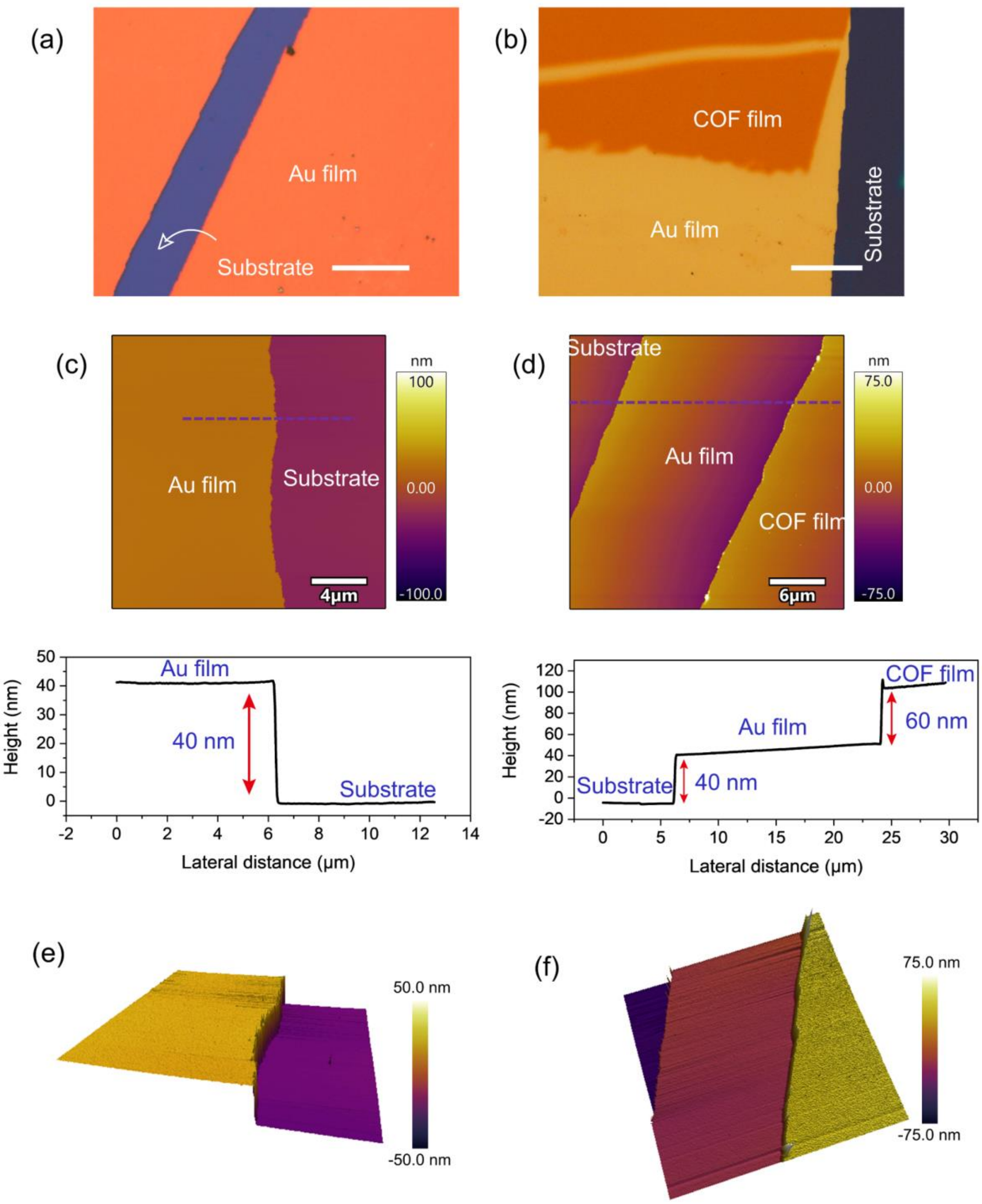

Supplementary Figure 11. OM and AFM images of PyTTA-TPA COF film grown on Au film. (a) OM image of the Au film deposited on a SiO_2/Si substrate. (b) OM image of the PyTTA-TPA COF film on the Au film. (c) AFM image of the Au film. The thickness of the Au film is about 40 nm. (d) AFM image of the PyTTA-TPA COF film on the Au film. The thickness of the PyTTA-TPA COF film is about 60 nm. (e, f) Corresponding three-dimensional shape of (c) and (d).

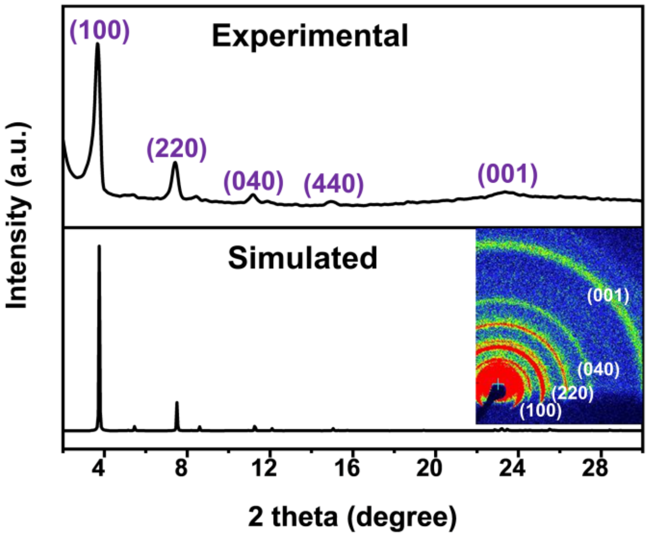

Supplementary Figure 12. GIWAXS and simulated XRD of PyTTA-TPA COF film grown on Au film with a thickness of 60 nm. The inset showed a 2D-GIWAXS image of PyTTA-TPA COF film. The 2D GIWAXS showed an arc pattern, and no orientation was found in the COF film[1], which indicated the crystallization process of irregular polygons to layered frameworks with ordered pores was driven by π–π stacking interactions of adjacent sheets[10].

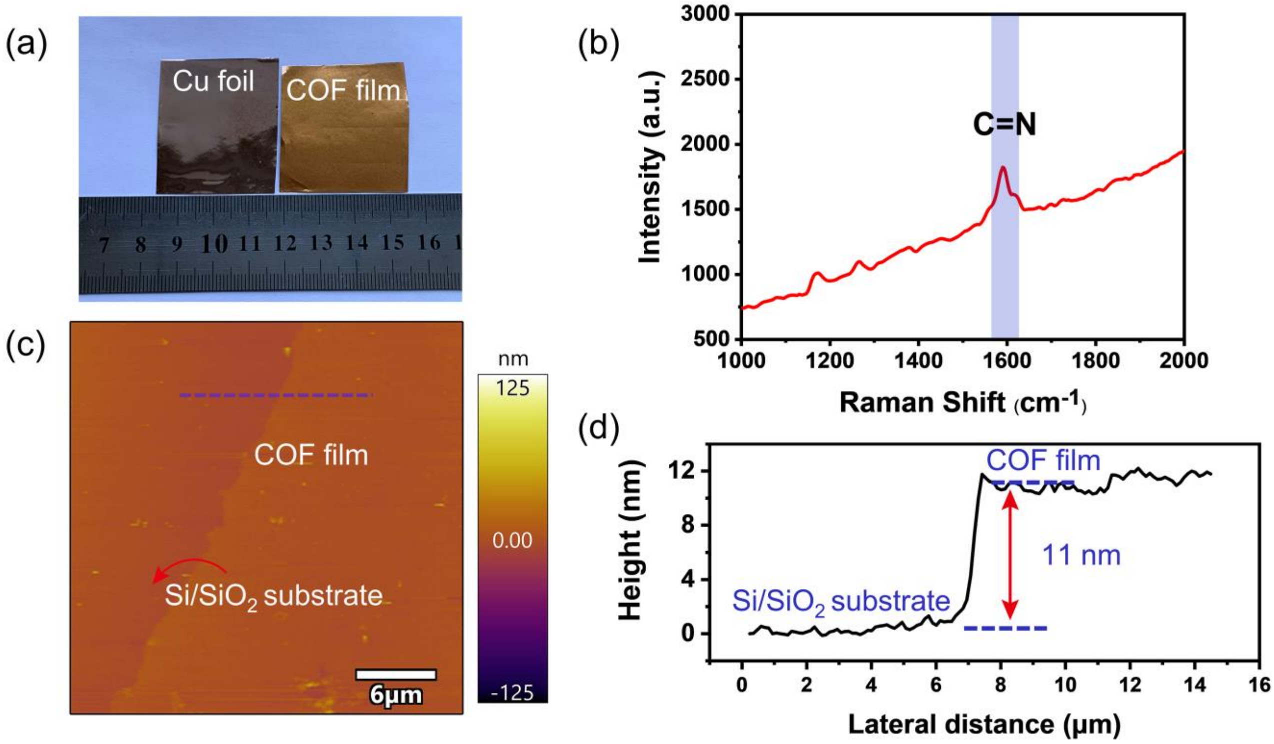

Supplementary Figure 13. PyTTA-TPA COF film grown on Cu foil. (a) OM image of Cu foil and PyTTA-TPA COF film grown on Cu foil. The clear color contrast indicated that the Cu foil (right) have been modified by COF film. (b) Raman spectra of PyTTA-TPA COF film grown on Cu foil. The appearance of new strong peak at 1618 cm^{-1} correspond to the stretching vibrations of the imine moieties, indicating the formation of the imine bond linking COF film on Cu foil. (c, d) AFM image and cross-section contour of the PyTTA-TPA COF film transferred onto Si/SiO_2 substrate. The thickness of the PyTTA-TPA COF film is about 11 nm.

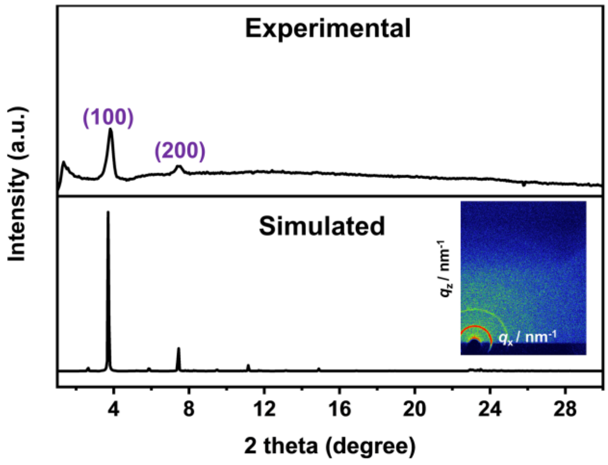

Supplementary Figure 14. GIWAXS and simulated XRD of PyTTA-TPA COF film grown on Cu substrate. The inset showed a 2D-GIWAXS image of PyTTA-TPA COF film.

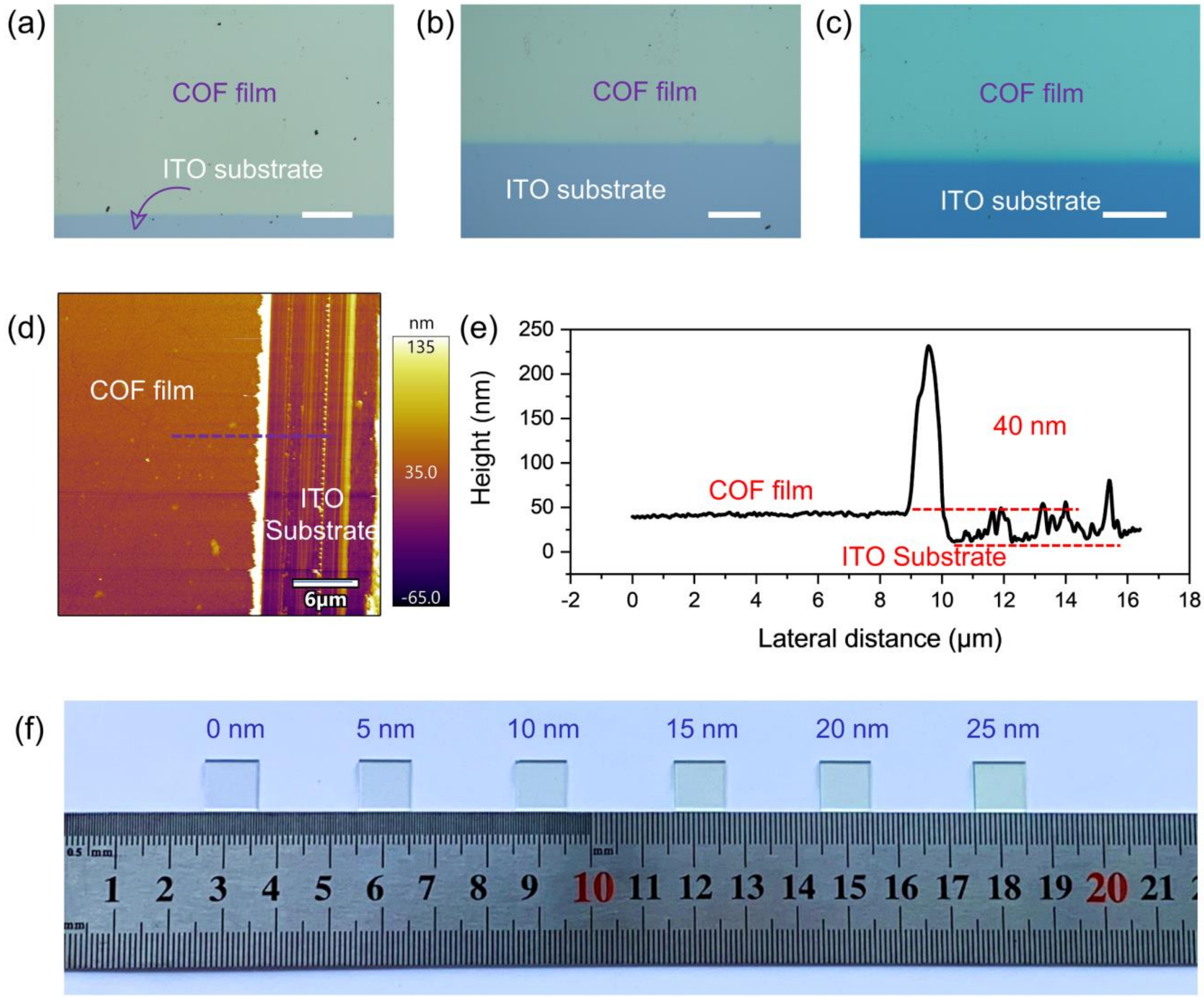

Supplementary Figure 15. PyTTA-TPA COF films grown on ITO conductive glass. (a–c) OM images of the PyTTA-TPA COF film on ITO conductive glass with different magnification. Scale bar 200 µm, 100 µm, 50 µm in (a), (b) and (c), respectively. (d, e) AFM image and cross-section contour of the PyTTA-TPA COF film. (f) Photograph of PyTTA-TPA COF films with different thickness.

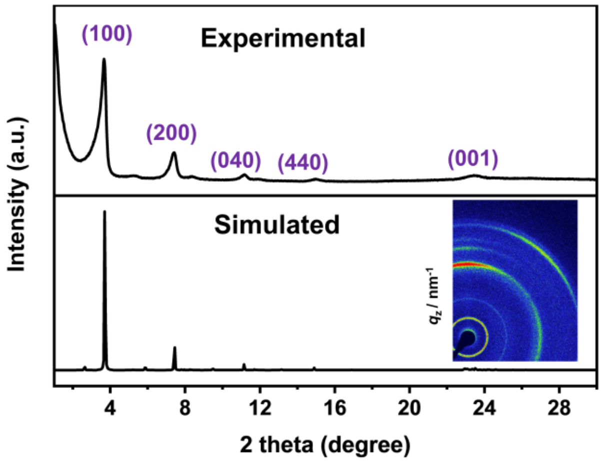

Supplementary Figure 16. GIWAXS and simulated XRD of PyTTA-TPA COF film grown on ITO film. The inset showed a 2D-GIWAXS image of PyTTA-TPA COF film.

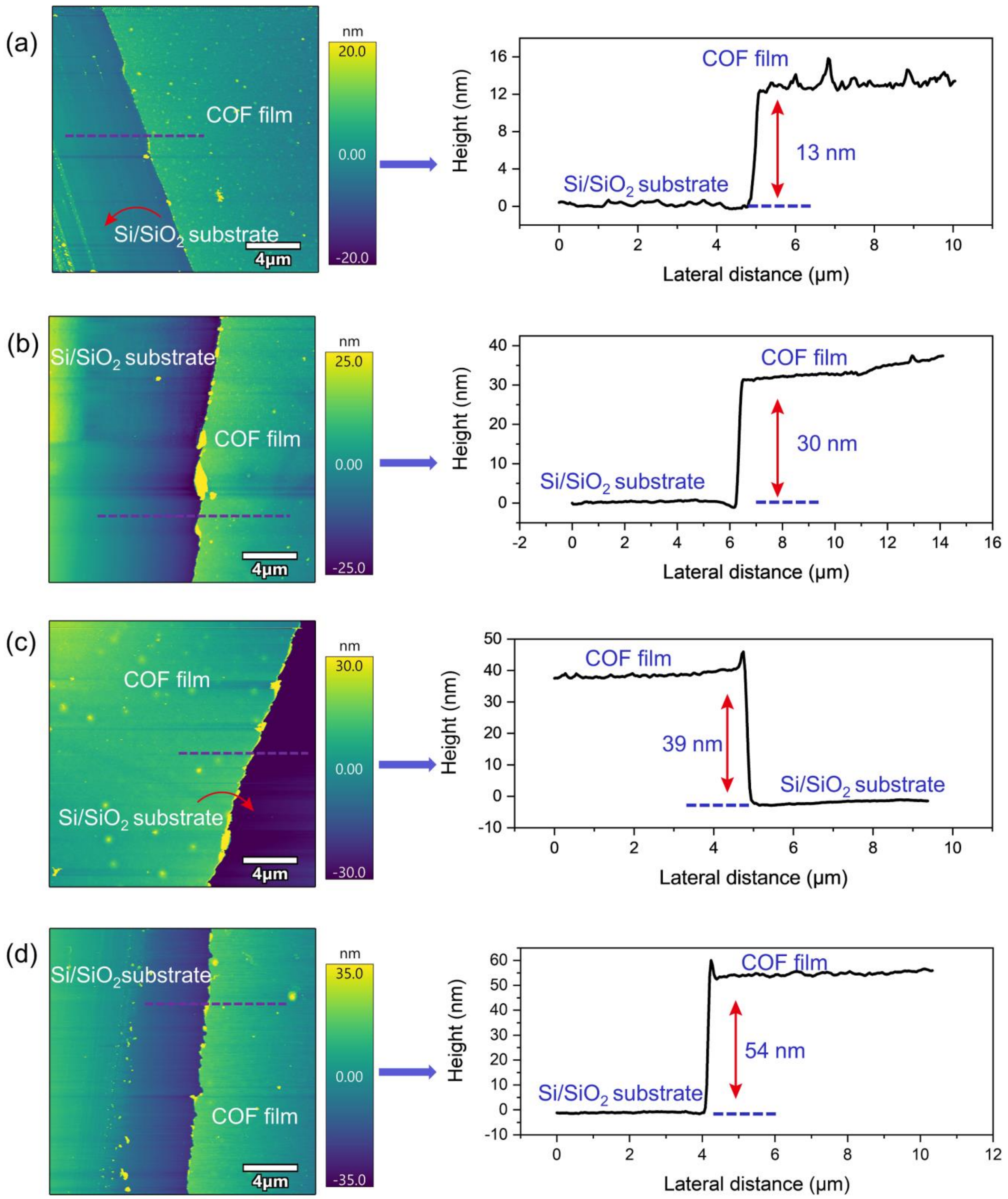

Supplementary Figure 17. AFM images of PyTTA-TPA COF films with different thickness. (a) 13 nm. (b) 30 nm. (c) 39 nm. (d) 54 nm.

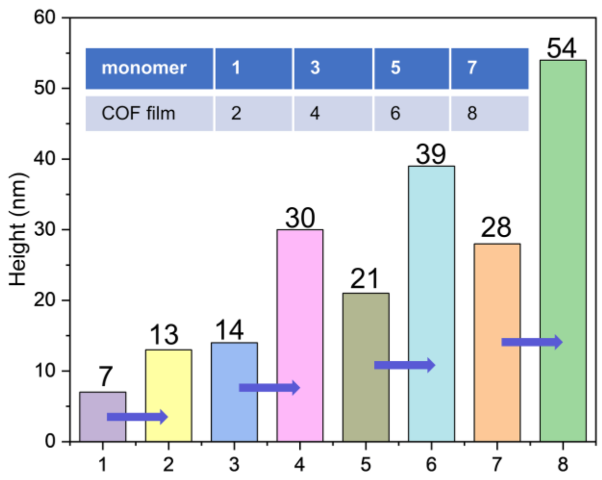

Supplementary Figure 18. Change of film thickness before and after growth according the AFM images showed in Supplementary Figure 3 and 17. The thickness of PyTTA-TPA COF films after polycondensation is about twice the thickness of the corresponding PyTTA monomer films, indicating the thickness of COFs films could be adjusted by controlling the thickness of PyTTA monomer.

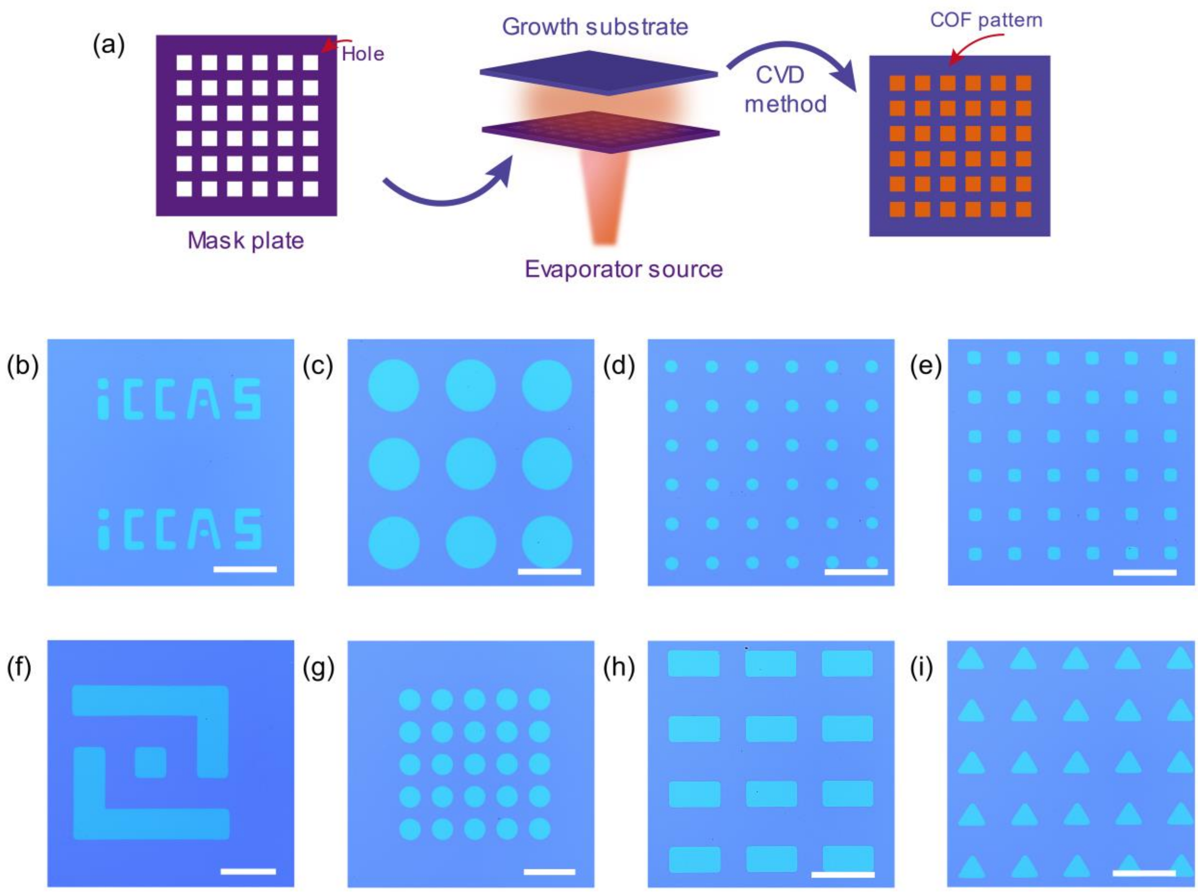

Supplementary Figure 19. PyTTA patterns prepared via different mask. (a) Schematic diagram of the preparation process of PyTTA patterns via thermal evaporation. (b–i) OM images of typical PyTTA patterns deposited on SiO_2/Si substrates with a thickness of ~14 nm. Scale bar 100 μm.

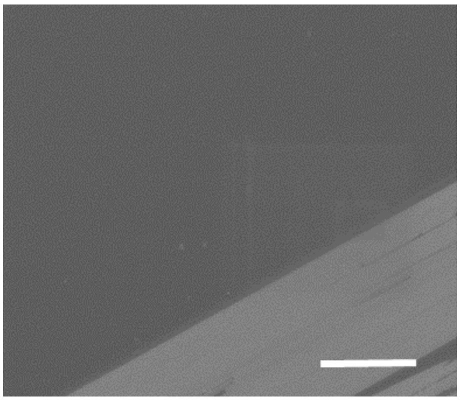

Supplementary Figure 20. SEM image of the PyTTA-TPA COF film. Scale bar 50 μm.

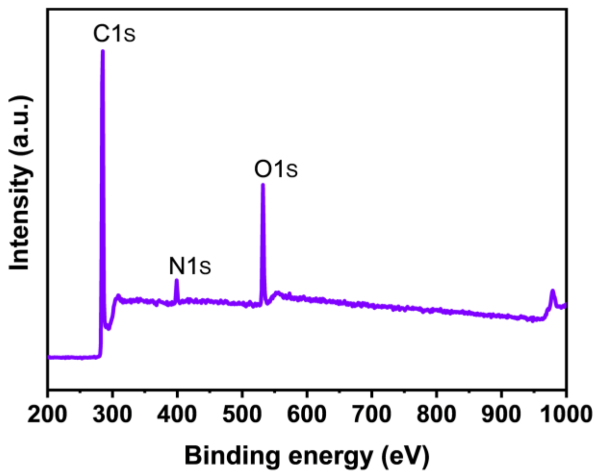

Supplementary Figure 21. XPS survey spectrum of the PyTTA-TPA COF film. The thickness of the PyTTA-TPA COF film is about 30 nm.

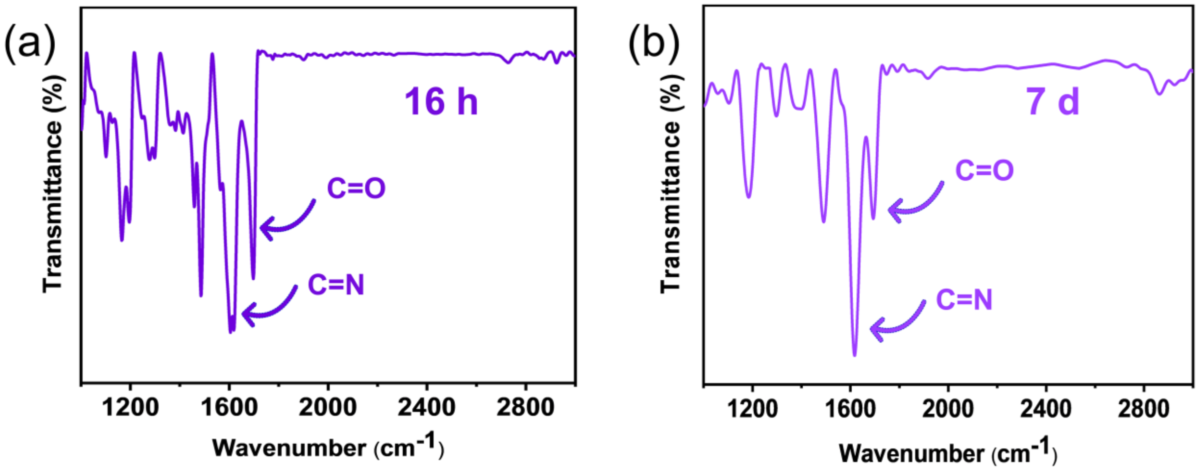

Supplementary Figure 22. FTIR spectra of PyTTA-TPA COF film grown with different reaction times. (a) The reaction time is 16 hours. (b) The reaction time is 7 days. Relative peak intensity ($I_{C=N}/I_{C=O}$) increases with the increase of reaction time, suggesting that the condensation reaction between amines and ketones is enhanced with the extension of reaction time.

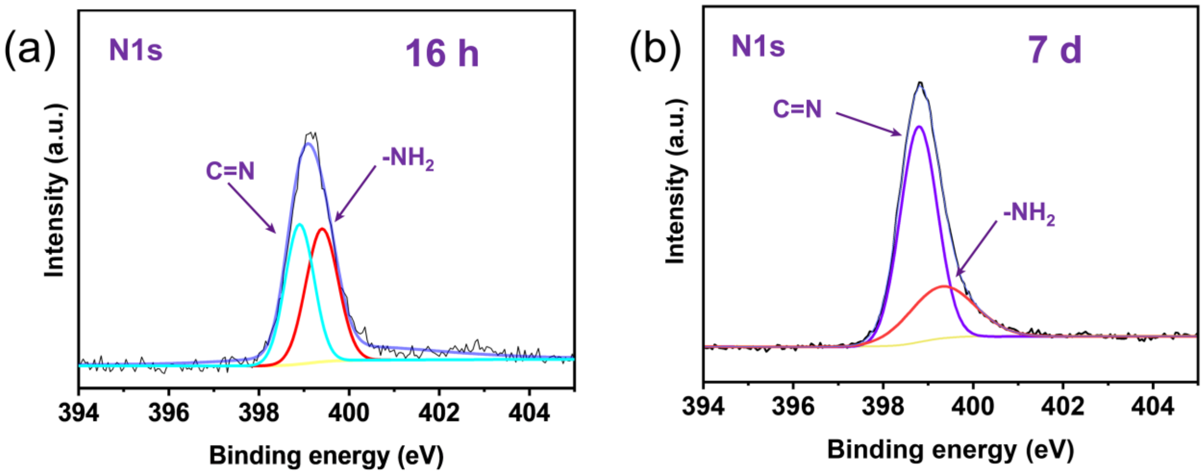

Supplementary Figure 23. N 1*s* XPS spectra of PyTTA-TPA COF film grown with different reaction times. (a) The reaction time is 16 hours. The intensity ratio of C=N and –NH_2 calculated from integrated area is 56.3: 43.7. (b) The reaction time is 7 days, the intensity ratio of C=N and –NH_2 calculated from integrated area is 72.9: 27.1. The integral range is 397 eV to 401 eV.

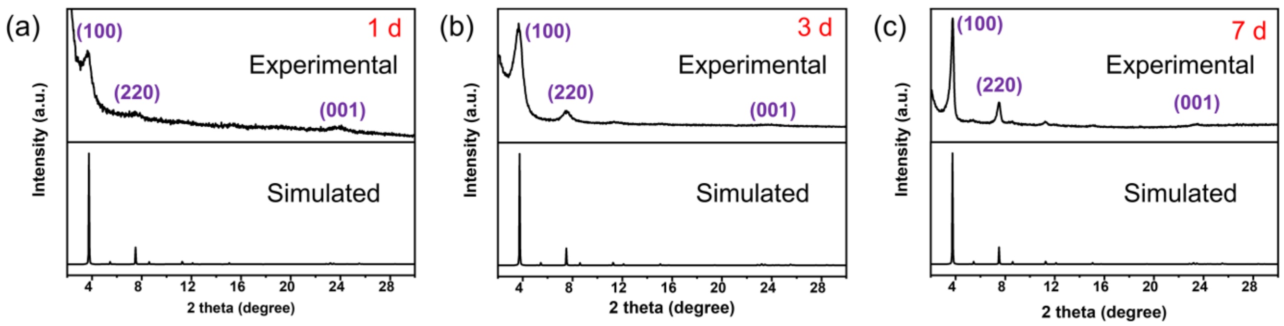

Supplementary Figure 24. GIWAXS of PyTTA-TPA COF films grown on SiO_2/Si substrate under different reaction time. (a) 1 day. (b) 3 day. (c) 7 day. The full width at half maximum (FWHM) of the (100) and (200) peaks decreased with the prolonging of growth time indicates that the degree of crystallization was enhanced. The thickness of PyTTA film using for the growth of PyTTA-TPA COF films is about 21 nm. The change in the PXRD results is a result of unreacted precursor that had sufficient time to react, and random polymer formed in the initial stage dissolved and crystallized into layered frameworks and ordered pores.

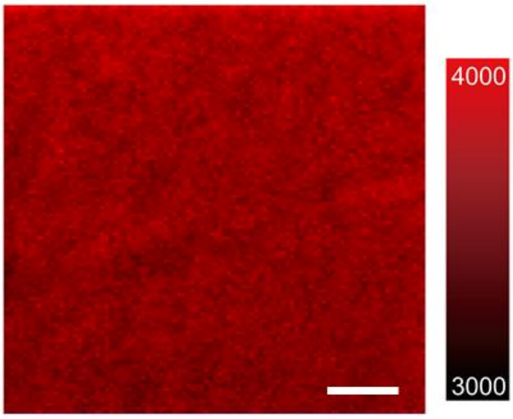

Supplementary Figure 25. Raman mapping image of a 2D COFs film at 1591 cm^{-1}. Scale bar 1 μm. The thickness of the PyTTA-TPA COF film is about 30 nm.

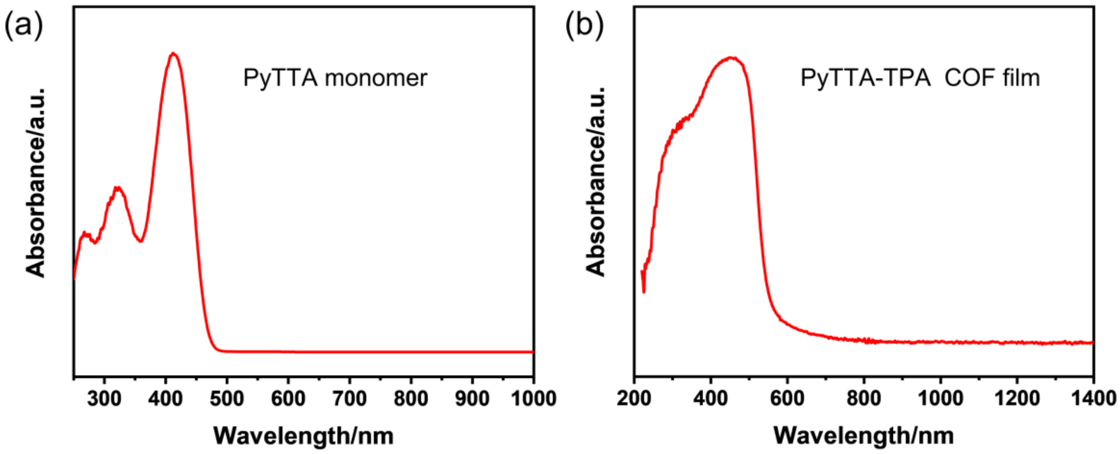

Supplementary Figure 26. UV–Vis absorption spectra of PyTTA monomer and PyTTA-TPA COF films. (a) PyTTA monomer film. (b) PyTTA-TPA COF film. The thickness of the PyTTA-TPA COF film is about 30 nm. Compared with PyTTA monomer, polymerization caused a redshift of UV-vis absorption spectrum.

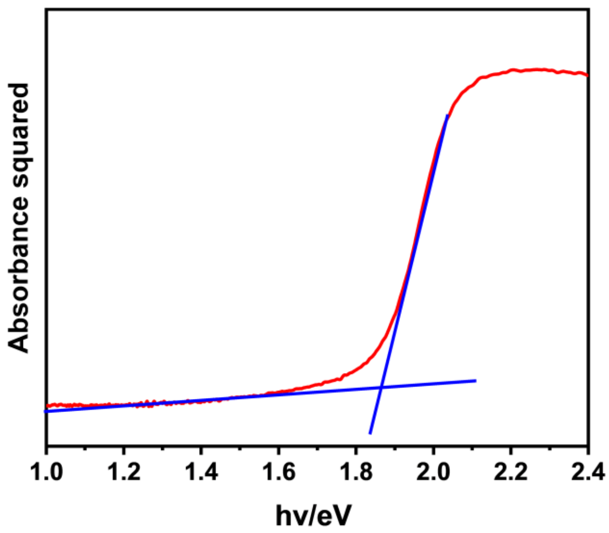

Supplementary Figure 27. Results of optical band-gap measurements and a plot of the absorbance squared vs. photon energy (*hv*) extrapolated to zero absorption.

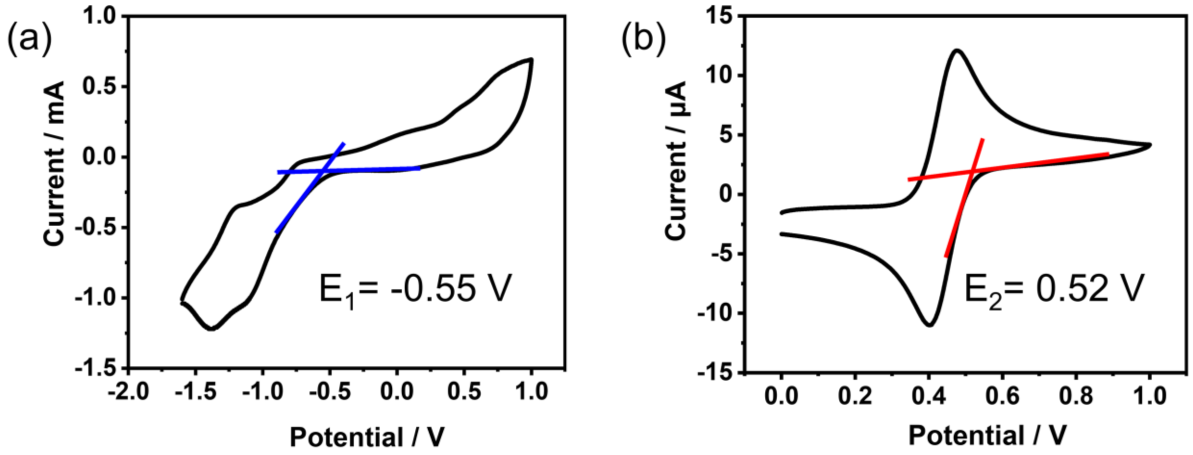

Supplementary Figure 28. Cyclic voltammograms (CV) in 0.1 M tetrabutylammonium hexafluorophosphate (TBAPF6) acetonitrile solution at a scan rate of 100 mV s^{-1} (Ag/AgCl as a reference electrode under N_2 atmosphere). (a) CV of the PyTTA-TPA COF film on GC electrode. (b) CV of the ferrocene.

The cyclic voltammograms (CV) were carried out to study the lowest unoccupied molecular orbital (LUMO) energy and high occupied molecular orbital (HOMO) energy level in a standard three electrode system, including working electrode, counter electrode (Platinum tablets), and reference electrode (Ag/AgCl). The supporting electrolyte is 0.1 M tetrabutylammonium hexafluorophosphate (TBAPF6) acetonitrile solution. And the LUMO energy level of the PyTTA-TPA COF film was calculated as following:

Fe/Fe^{+} (vs. Ag/AgCl) is 0.52 V,

E_1 (vs. Ag/AgCl) is –0.55 V,

LUMO (eV): $-4.8 - (E_1 - Fe/Fe^{+}) = -4.8 - (-0.55 - 0.52) = -3.73$ eV.

The LUMO energy level is experimentally estimated by onset of the redox potentials taking the known reference level for ferrocene, where 4.8 eV below the vacuum level according to the related equation[11,12].

The optical band-gap of PyTTA-TPA COF is 1.87 eV (Supplementary Figure 19),

HOMO (eV): –3.73 eV – 1.87 eV = –5.6 eV.

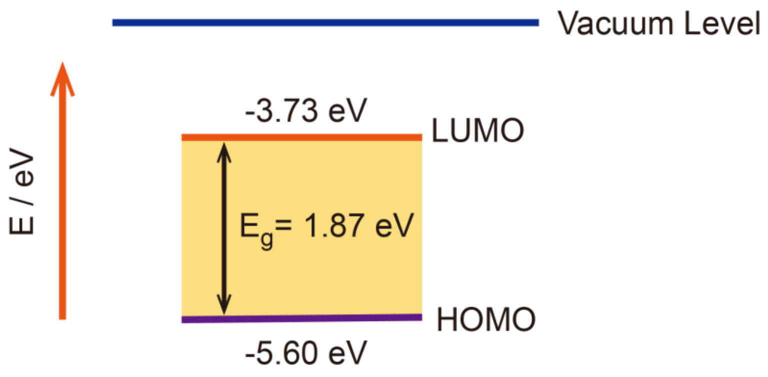

Supplementary Figure 29. Energy level diagram of the PyTTA-TPA COF film.

Supplementary Table 1. Atomic coordinates of the AA-stacking mode of PyTTA-TPA COF film using DFTB+ method.

Space group: C2/m<br>a = 24.5 Å, b = 24.1 Å and c = 4.3 Å.<br>α = 90°, β = 91° and γ = 90°				
	**Atom**	**X**	**Y**	**Z**
1)	C1	0.591340065	0.5970302	0.64858
2)	C2	0.537250042	0.6124702	0.7017
3)	C3	0.496079922	0.5714302	0.70413
4)	C4	0.508590221	0.51686	0.60955
5)	C5	0.563079834	0.50247	0.53742
6)	C6	0.605209827	0.5423102	0.57514
7)	C7	0.466380119	0.4766998	0.58514
8)	C8	0.478909969	0.4220901	0.49142
9)	C9	0.532299995	0.4098701	0.39778
10)	C10	0.573599815	0.4491601	0.42178
11)	C11	0.442709923	0.58359	0.79854
12)	C12	0.401370049	0.5443602	0.77341
13)	C13	0.41189003	0.4910998	0.6571
14)	C14	0.369740009	0.4513001	0.61912
15)	C15	0.383619785	0.3965602	0.54637
16)	C16	0.437739849	0.3810501	0.49442
17)	C17	0.450809956	0.3211498	0.48504
18)	C18	0.310800076	0.4653201	0.64112
19)	C19	0.664159775	0.5283098	0.55286
20)	C20	0.524169922	0.6723299	0.71559
21)	C21	0.419020176	0.2841101	0.30561
22)	C22	0.428919792	0.2271099	0.32284
23)	C23	0.471049786	0.2067299	0.51997
24)	C24	0.503240108	0.2437902	0.69235
25)	C25	0.492690086	0.3002901	0.67991

26	C26	0.288609982	0.50913	0.46384
27	C27	0.23251009	0.5191998	0.46001
28	C28	0.197199821	0.4852901	0.62815
29	C29	0.219349861	0.44174	0.81034
30	C30	0.275650024	0.4319901	0.81628
31	C31	0.686389923	0.48455	0.73028
32	C32	0.742479801	0.4744301	0.73327
33	C33	0.777729988	0.5082202	0.56411
34	C34	0.755559921	0.5518599	0.38267
35	C35	0.699269772	0.5616498	0.37744
36	C36	0.555699825	0.70889	0.89958
37	C37	0.545060158	0.76582	0.89152
38	C38	0.502419949	0.7865701	0.69941
39	C39	0.470870018	0.74999	0.51996
40	C40	0.482150078	0.6936002	0.52321
41	C41	0.100120068	0.46766	0.71997
42	C42	0.87473011	0.5247698	0.46408
43	C43	0.516550064	0.8857002	0.79102
44	C44	0.453420162	0.1075501	0.45867
45	C45	0.499509811	0.9423599	0.71107
46	C46	0.932240009	0.5096202	0.52965
47	C47	0.042630196	0.4828601	0.65455
48	C48	0.469719887	0.0509601	0.54508
49	C49	0.529809952	0.9873199	0.82663
50	C50	0.453969955	0.9520702	0.5131
51	C51	0.946040154	0.4643102	0.72251
52	C52	0.974110126	0.5413699	0.40008
53	C53	0.439219952	0.0060201	0.43122
54	C54	0.515100002	0.0412798	0.74438
55	C55	0.028820038	0.5281901	0.46177
56	C56	0.000750065	0.4510798	0.7839

57	N1	0.481790066	0.1490202	0.56441
58	N2	0.139560223	0.4958301	0.59738
59	N3	0.49048996	0.8442798	0.66668
60	N4	0.83536005	0.4975801	0.59374
61	H1	0.62308979	0.6283202	0.6596
62	H2	0.54279995	0.3696299	0.30532
63	H3	0.613790035	0.4372902	0.34651
64	H4	0.432250023	0.6237202	0.89264
65	H5	0.361179829	0.5562	0.84871
66	H6	0.351850033	0.3652802	0.5358
67	H7	0.386539936	0.2993002	0.15304
68	H8	0.403709888	0.1998501	0.18143
69	H9	0.535369873	0.2284999	0.84703
70	H10	0.516560078	0.3276801	0.8286
71	H11	0.314589977	0.53473	0.32188
72	H12	0.216179848	0.5526199	0.31726
73	H13	0.193999767	0.4148598	0.94763
74	H14	0.291790009	0.3980899	0.95623
75	H15	0.660470009	0.4590302	0.87298
76	H16	0.758840084	0.441	0.87592
77	H17	0.780889988	0.5788398	0.24589
78	H18	0.683100224	0.5955901	0.23792
79	H19	0.588389874	0.6933699	0.04954
80	H20	0.570119858	0.7927098	0.0361201
81	H21	0.4384799	0.7656102	0.36818
82	H22	0.45855999	0.6666198	0.37067
83	H23	0.108310223	0.4319601	0.86612
84	H24	0.866489887	0.5595698	0.31138
85	H25	0.551859856	0.87958	0.94238
86	H26	0.416959763	0.1136799	0.31671
87	H27	0.564879894	0.9807	0.98014

**88**	**H28**	**0.429920197**	**0.9180398**	**0.4191**
**89**	**H29**	**0.914649963**	**0.4391799**	**0.8272**
**90**	**H30**	**0.964489937**	**0.5763502**	**0.24993**
**91**	**H31**	**0.404230118**	**0.01264**	**0.27712**
**92**	**H32**	**0.539179802**	**0.0753198**	**0.83799**
**93**	**H33**	**0.060210228**	**0.5533099**	**0.357**
**94**	**H34**	**0.010369778**	**0.41608**	**0.9339**

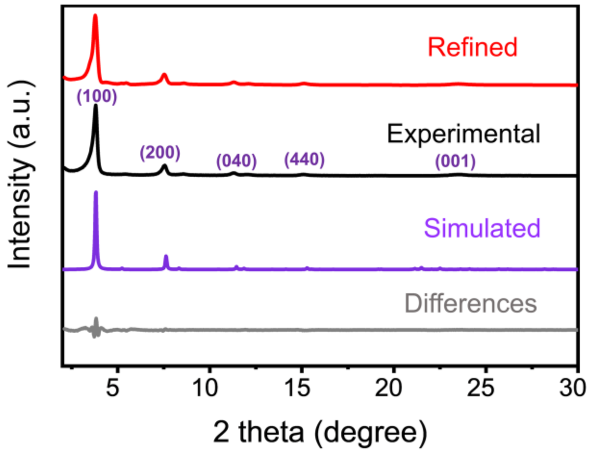

Supplementary Figure 30. PXRD characterization results of PyTTA-TPA COF powder via the solvothermal method. The crystal size of the PyTTA-TPA COFs is ~22.0 nm by Scherrer's analysis.

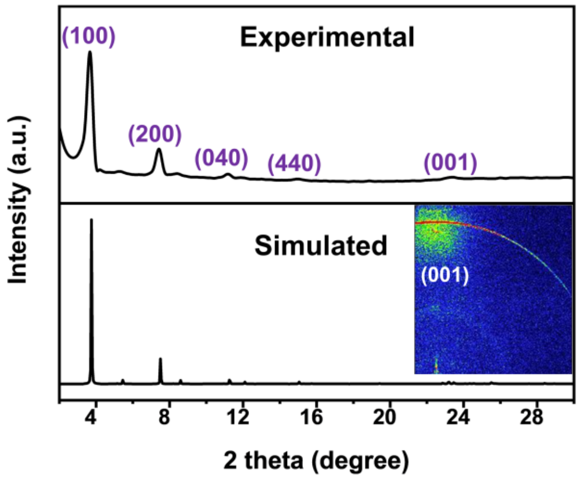

Supplementary Figure 31. GIWAXS and simulated XRD of PyTTA-TPA COF film grown on highly oriented pyrolytic graphite (HOPG) substrate with a thickness of 30 nm. The inset showed a 2D-GIWAXS image of PyTTA-TPA COF film. The drastic change in intensity of the (001) signal in the in-plane (q_x) and out-of-plane (q_z) directions proves the formation of oriented COF crystalline regions that preferably lie with the *ab* plane parallel to the HOPG surface[13,14].

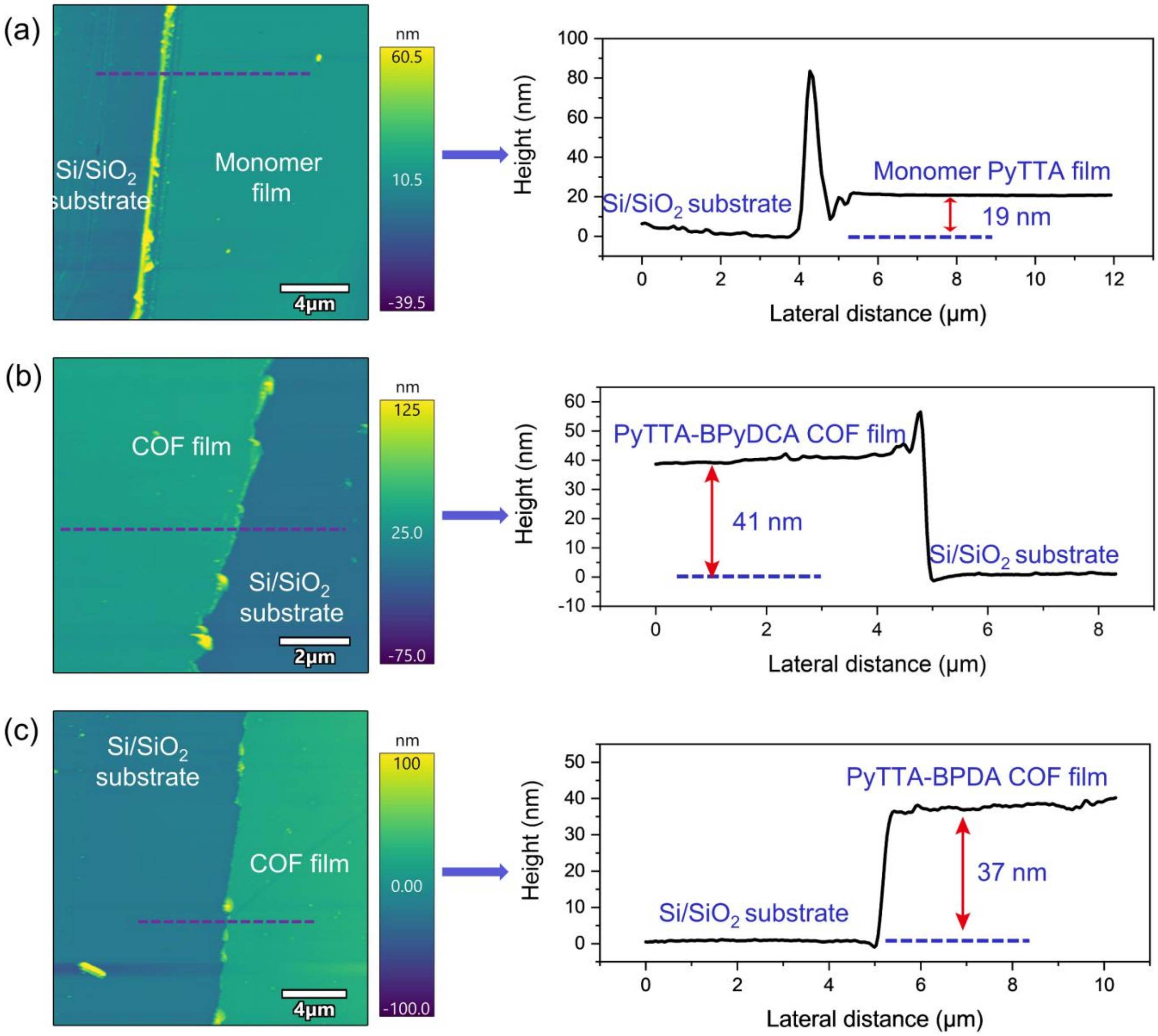

Supplementary Figure 32. AFM images of PyTTA-BPyDCA and PyTTA-BPDA COF films. (a) AFM image of PyTTA film using for the growth of PyTTA-BPyDCA and PyTTA-BPDA COF films. (b) AFM image of PyTTA-BPyDCA COF film after growth. (c) AFM image of PyTTA-BPDA COF film after growth.

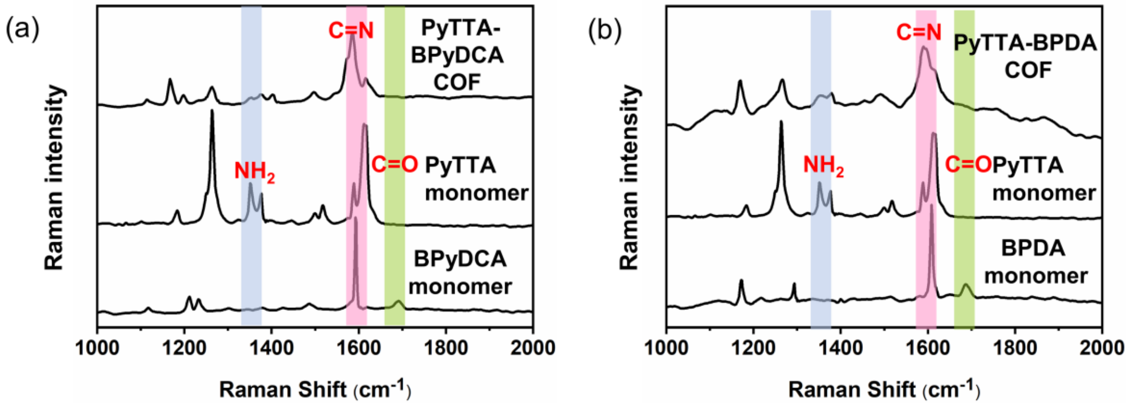

Supplementary Figure 33. Raman spectra of PyTTA-BPyDCA and PyTTA-BPDA COF films. (a) PyTTA-BPyDCA COF film and its corresponding monomer. The thickness of the PyTTA-BPyDCA COF film is about 41 nm. (b) PyTTA-BPDA COF film and its corresponding monomer. The thickness of the PyTTA-BPDA COF film is about 37 nm.

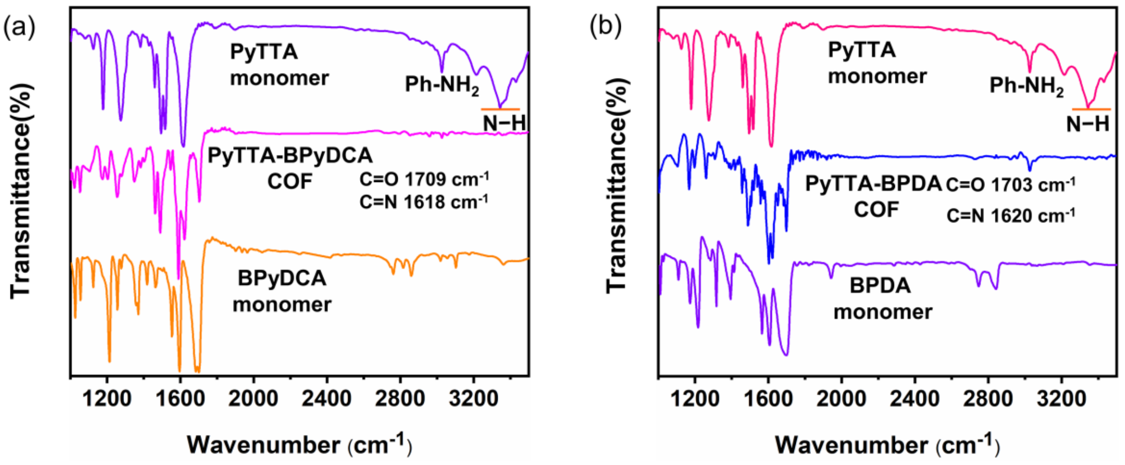

Supplementary Figure 34. FTIR spectra of PyTTA-BPyDCA and PyTTA-BPDA COF films. (a) Infrared spectra of PyTTA-BPyDCA COF film and its corresponding monomer. The thickness of the PyTTA-BPyDCA COF film is about 41 nm. (b) Infrared spectra of PyTTA-BPDA COF film and its corresponding monomer. The thickness of the PyTTA-BPDA COF film is about 37 nm. Adsorption bands of the $-NH_2$ groups of PyTTA located at ~3330 and 3230 cm^{-1} disappear after growth with the subsequent appearance of a peak at ~1620 cm^{-1}, corresponding to the C=N stretching modes characteristic of imines, indicating the formation of –C=N– linkages in PyTTA-BPyDCA and PyTTA-BPDA COF films.

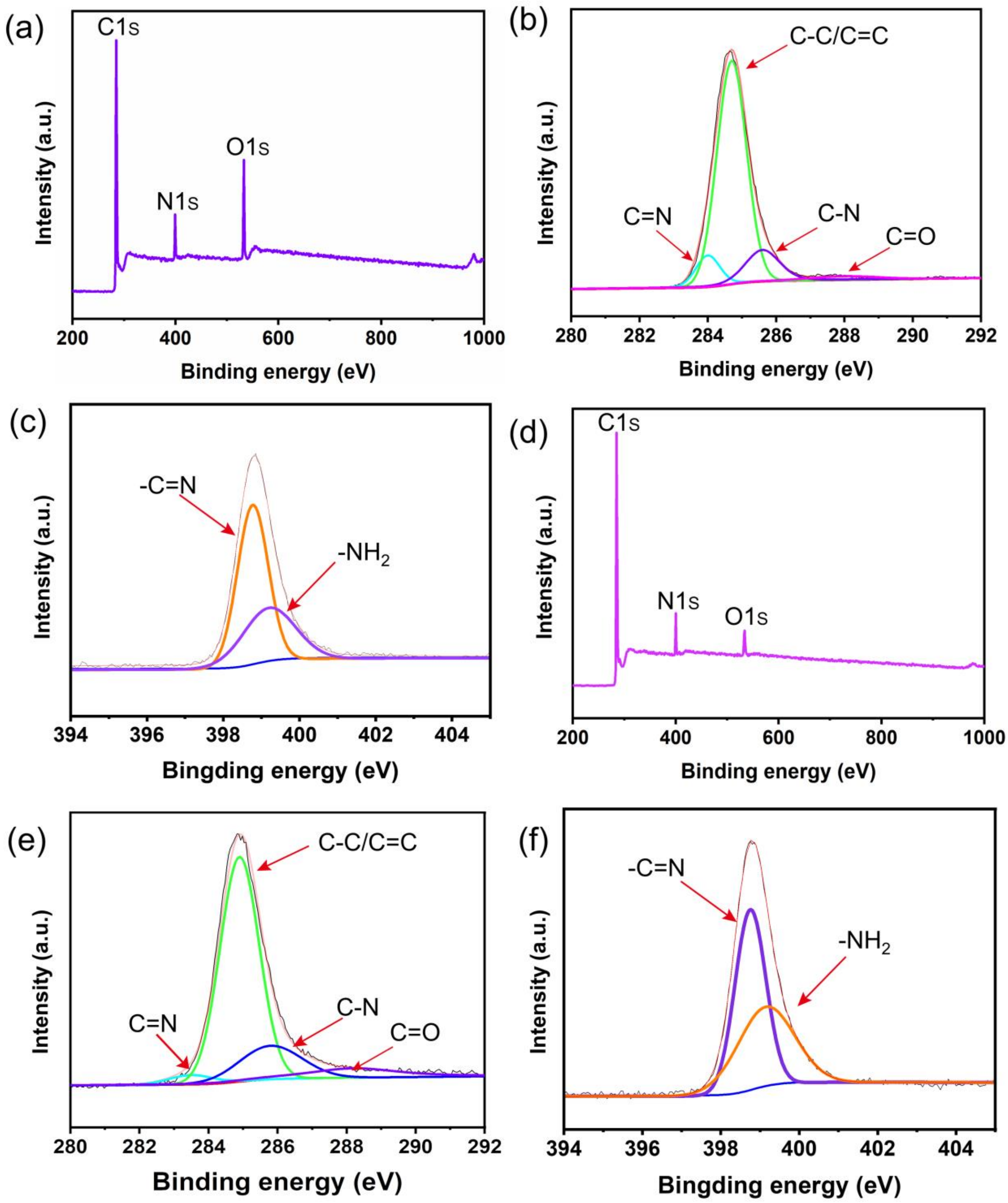

Supplementary Figure 35. XPS spectra of PyTTA-BPyDCA and PyTTA-BPDA COF films. (a) The full XPS spectrum of PyTTA-BPyDCA COF film. (b) C 1*s* XPS spectrum of the PyTTA-BPyDCA COF film. (c) N 1*s* XPS spectrum of the PyTTA-BPyDCA COF film. (d) The full XPS spectrum of PyTTA-BPDA COF film. (e) C 1*s* XPS spectrum of the PyTTA-BPDA COF film. (f) N 1*s* XPS spectrum of the PyTTA-BPDA COF film. The thickness of the PyTTA-BPyDCA COF film is about 41 nm. The thickness of the PyTTA-BPDA COF film is about 37 nm.

Supplementary Table 2. Atomic coordinates of the AA-stacking mode of PyTTA-BPyDCA COF using DFTB+ method.

Space group: C2/m<br>a = 42.6 Å, b = 36.7 Å and c = 4.4 Å.<br>α = 90°, β = 90.5 °and γ = 90°				
	**Atom**	**X**	**Y**	**Z**
1)	N1	0.3531	0.3466	0.1754
2)	C2	0.4371	0.467	0.2713
3)	C3	0.4687	0.4666	0.3803
4)	C4	0.4846	0.434	0.443
5)	C5	0.4168	0.4346	0.2512
6)	C6	0.4238	0.4058	0.0505
7)	C7	0.4031	0.3762	0.0276
8)	C8	0.3753	0.3755	0.2045
9)	C9	0.3683	0.4042	0.403
10)	C10	0.3887	0.4336	0.426
11)	C11	0.3483	0.3241	0.4004
12)	C12	0.3203	0.3004	0.401
13)	C13	0.2933	0.3094	0.2289
14)	C14	0.319	0.2707	0.6015
15)	H15	0.4734	0.408	0.4057
16)	H16	0.4449	0.4066	-0.0912
17)	H17	0.4083	0.3543	-0.1294
18)	H18	0.3467	0.4035	0.5388
19)	H19	0.383	0.4555	0.5827
20)	C20	0.2655	0.2894	0.2651
21)	C21	0.2648	0.2604	0.4739
22)	N22	0.2917	0.2517	0.6328
23)	H23	0.3632	0.3248	0.6044
24)	H24	0.2936	0.3323	0.0725
25)	H25	0.3395	0.2634	0.7401
26)	H26	0.2446	0.2967	0.1332
27)	C27	0.4221	0.5	0.2209
28)	C28	0.4844	0.5	0.438

Supplementary Table 3. Atomic coordinates of the AA-stacking mode of PyTTA-BPDA COF using DFTB+ method.

Space group: C2/m<br>a = 42.3 Å, b = 36.9 Å and c = 4.4 Å.<br>α = 90°, β = 90° and γ = 90°				
	Atom	X	Y	Z
1)	N1	0.3531	0.3466	0.1754
2)	C2	0.4371	0.467	0.2713
3)	C3	0.4687	0.4666	0.3803
4)	C4	0.4846	0.434	0.443
5)	C5	0.4168	0.4346	0.2512
6)	C6	0.4238	0.4058	0.0505
7)	C7	0.4031	0.3762	0.0276
8)	C8	0.3753	0.3755	0.2045
9)	C9	0.3683	0.4042	0.403
10)	C10	0.3887	0.4336	0.426
11)	C11	0.3483	0.3241	0.4004
12)	C12	0.3203	0.3004	0.401
13)	C13	0.2933	0.3094	0.2289
14)	C14	0.319	0.2707	0.6015
15)	H15	0.4722	0.407	0.4005
16)	H16	0.4464	0.4065	-0.094
17)	H17	0.4089	0.3529	-0.1351
18)	H18	0.3456	0.4035	0.547
19)	H19	0.3828	0.457	0.5875
20)	C20	0.2655	0.2894	0.2651
21)	C21	0.2648	0.2604	0.4739
22)	C22	0.2917	0.2517	0.6328
23)	H23	0.3658	0.3226	0.6005
24)	H24	0.2941	0.333	0.0593
25)	H25	0.341	0.2625	0.7389
26)	H26	0.2434	0.2966	0.1263
27)	H27	0.2916	0.2277	0.7987
28)	C28	0.4221	0.5	0.2209
29)	C29	0.4844	0.5	0.438

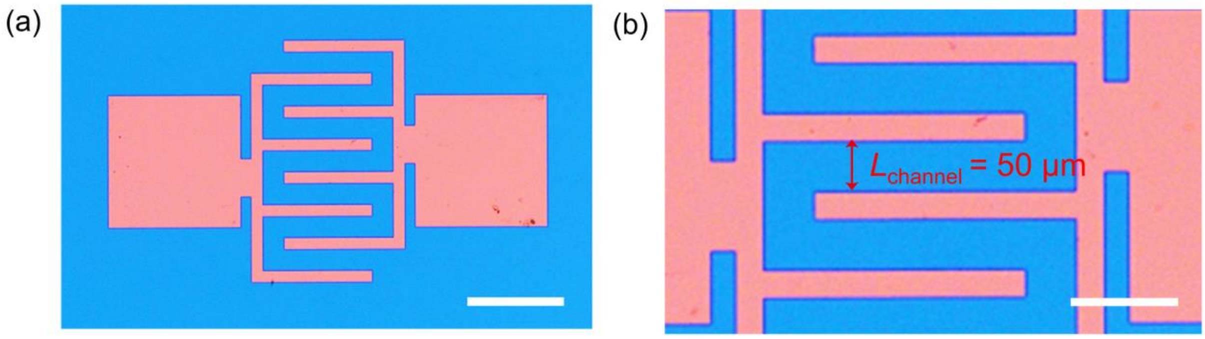

Supplementary Figure 36. OM images of a PyTTA-TPA-based FET. (a) Scale bar 200 μm. (b) Scale bar 100 μm.

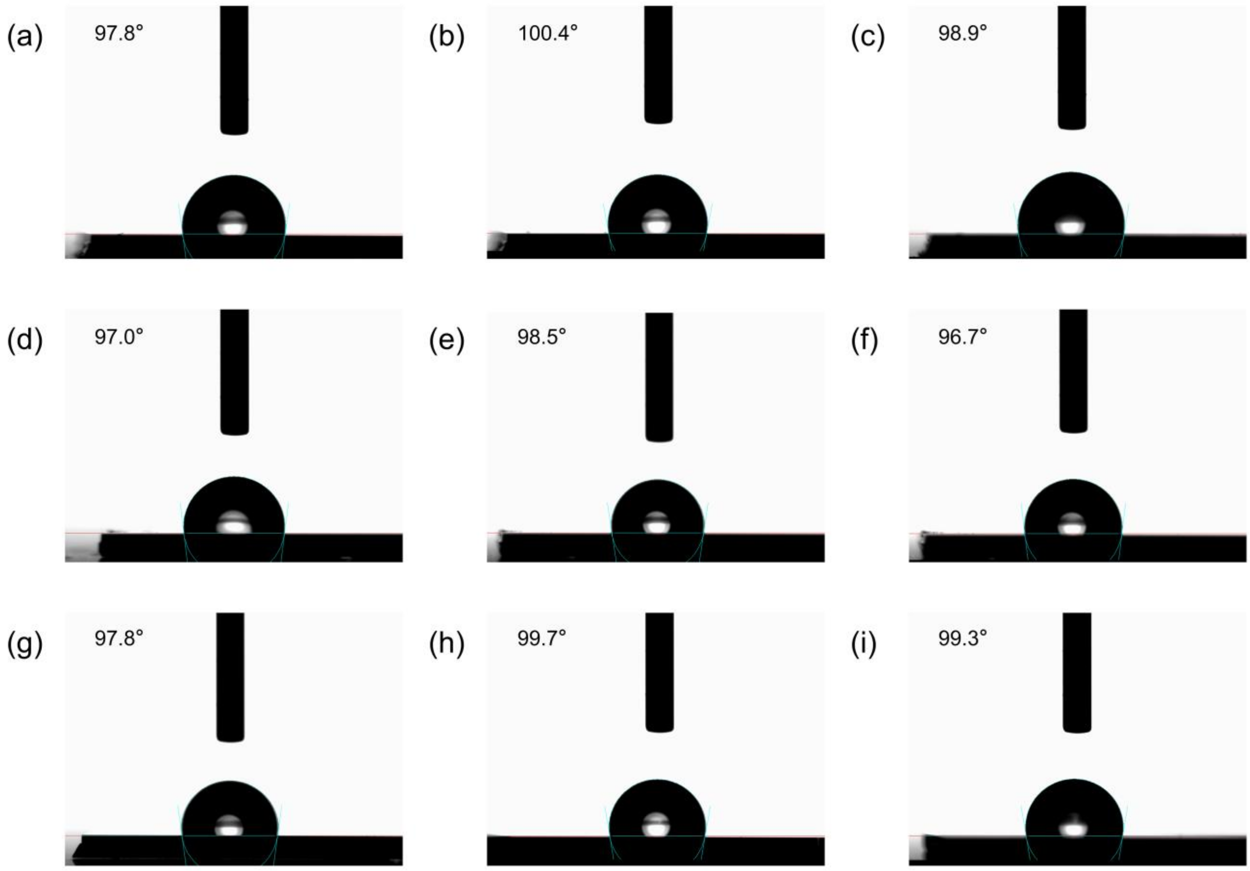

Supplementary Figure 37. Super-hydrophobicity of OTS-modified SiO_2/Si substrates. (a–c) Contact angle of OTS-modified SiO_2/Si substrates before annealing. (d–f) Contact angle of OTS-modified SiO_2/Si substrates after 1 hour vacuum treatment (6 x 10^{-6} mbar). (g–i) Contact angle of OTS-modified SiO_2/Si substrates after heat annealing. The heat annealing conditions are similar to that of COF growth. Three random areas were selected on each sample, and the water droplet volume was 2 microl throughout the measurements. The OTS-modified SiO_2/Si substrate still has a superhydrophobic surface after annealing, which confirms that the OTS coating is still anchored on SiO_2/Si surface after a COF growth.

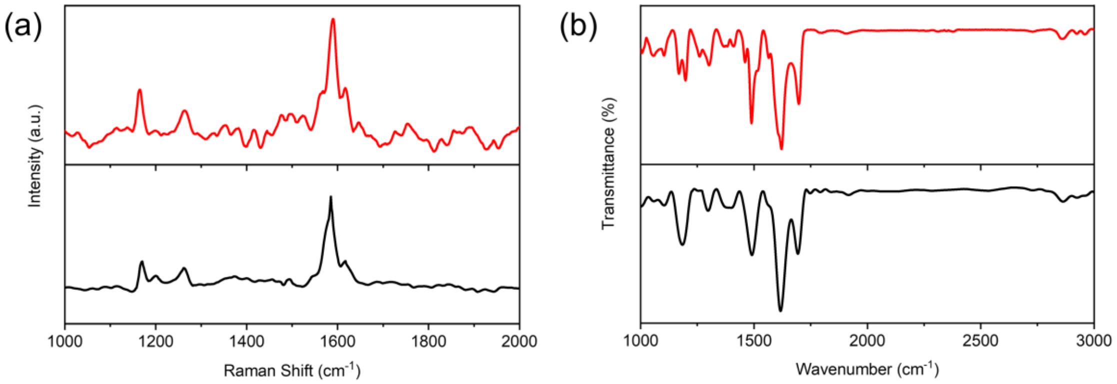

Supplementary Figure S38. Raman and IR characterization of PyTTA-TPA COF films grown on OTS-modified SiO_2/Si substrates. (a) Raman spectra of COF films grown on OTS-modified SiO_2/Si substrates (red line) and SiO_2/Si substrates (black line). (b) IR spectra of COF films grown on OTS-modified SiO_2/Si substrates (red line) and SiO_2/Si substrates (black line). Compared with that grown on SiO_2/Si substrates, the COF film on OTS-modified SiO_2/Si substrate is not influenced by the OTS.

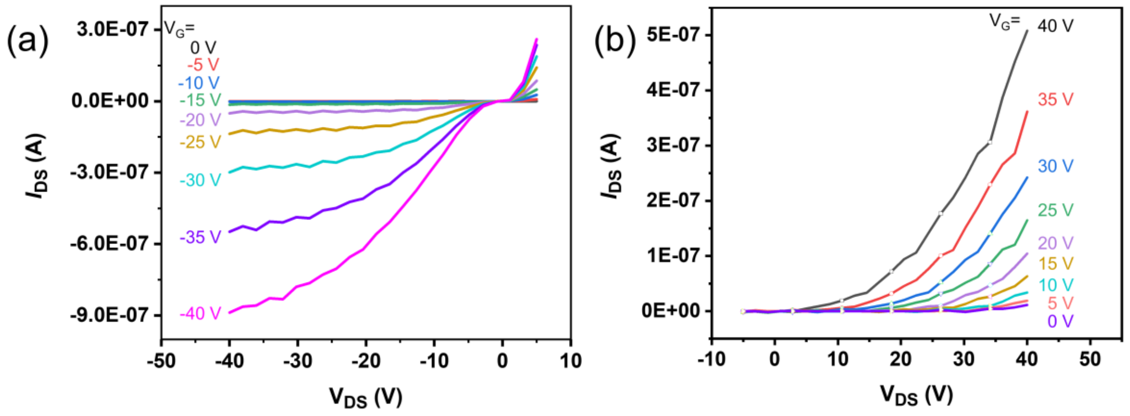

Supplementary Figure 39. I_{DS}–V_{DS} characteristics of PyTTA-TPA COF film-based FET devices. (a) I_{DS}–V_{DS} characteristics for negative V_{DS} voltage. (b) I_{DS}–V_{DS} characteristics for positive V_{DS} voltage. In Supplementary Figure 39a, I_{DS} increase with the increase of negative V_G, and in Figure S39b, I_{DS} decrease with the increase of positive V_G, indicative of *p*-type behavior.

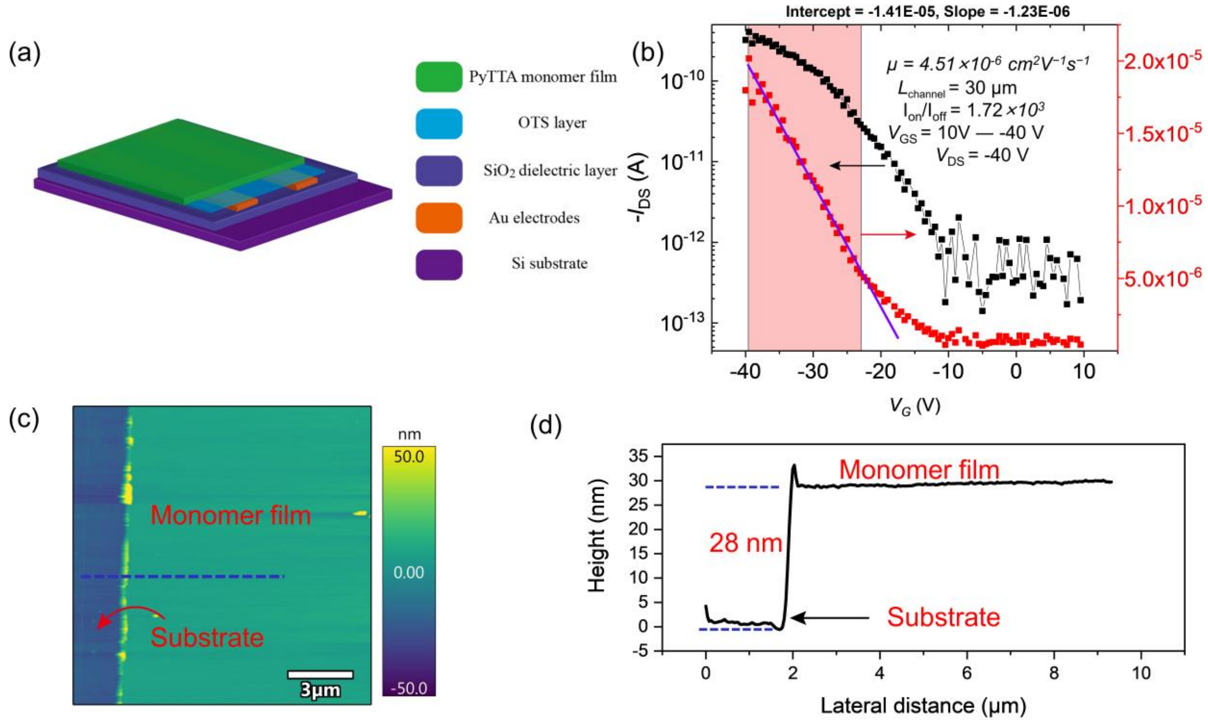

Supplementary Figure 40. Electrical characteristics of PyTTA film-based TFTs. (a) Schematic diagram of a FET device using PyTTA film as working materials. (b) Transfer characteristics (I_{DS} vs V_G) of the PyTTA based FET device at V_{DS} = –40 V. The transistor exhibits a *p*-channel behavior with a mobility of ~4.51 × 10^{-6} $cm^2\ V^{-1}\ s^{-1}$, and a high on/off ratio of 10^3. (c, d) AFM image and cross-section contour of the PyTTA film loaded on OTS-modified SiO_2/Si substrate by thermal evaporation for the FET device.

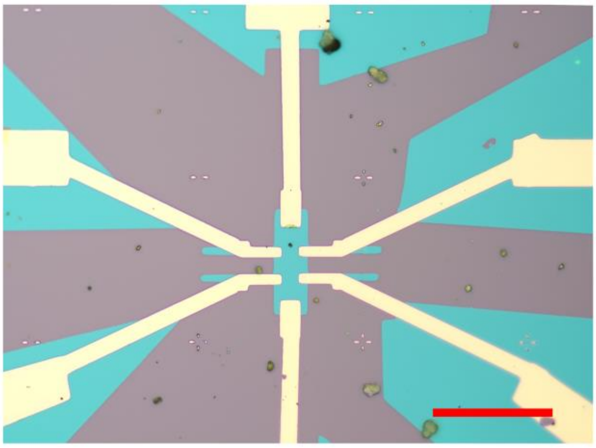

Supplementary Figure 41. Optical image of a typical Hall-effect device based on PyTTA-TPA COF film. Scale bar is 50 µm.

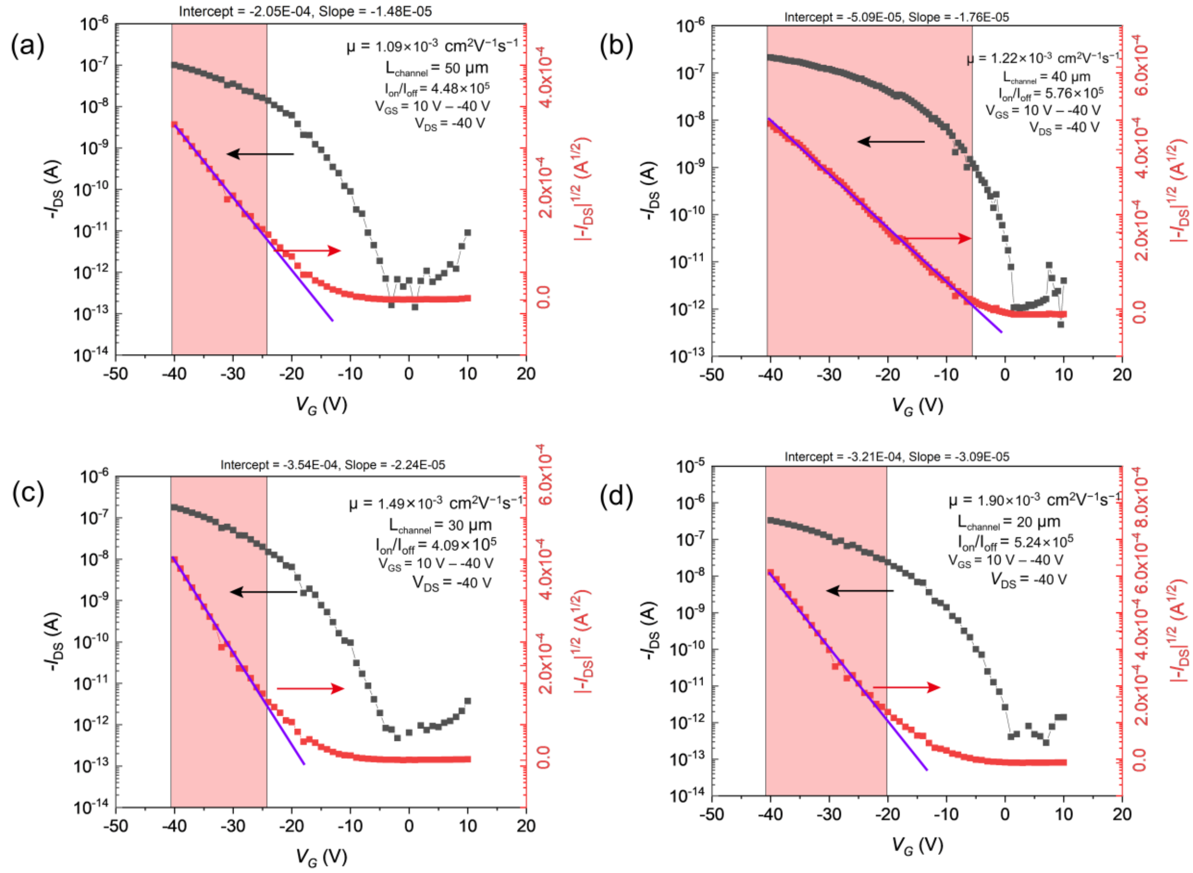

Supplementary Figure 42. Transfer characteristics (I_{DS} vs V_G) of the PyTTA-TPA COF device with different channel lengths at V_{DS} = –40 V. (a) Channel length is ~50 µm. (b) Channel length is ~40 µm. (c) Channel length is ~30 µm. (d) Channel length is ~20 µm.

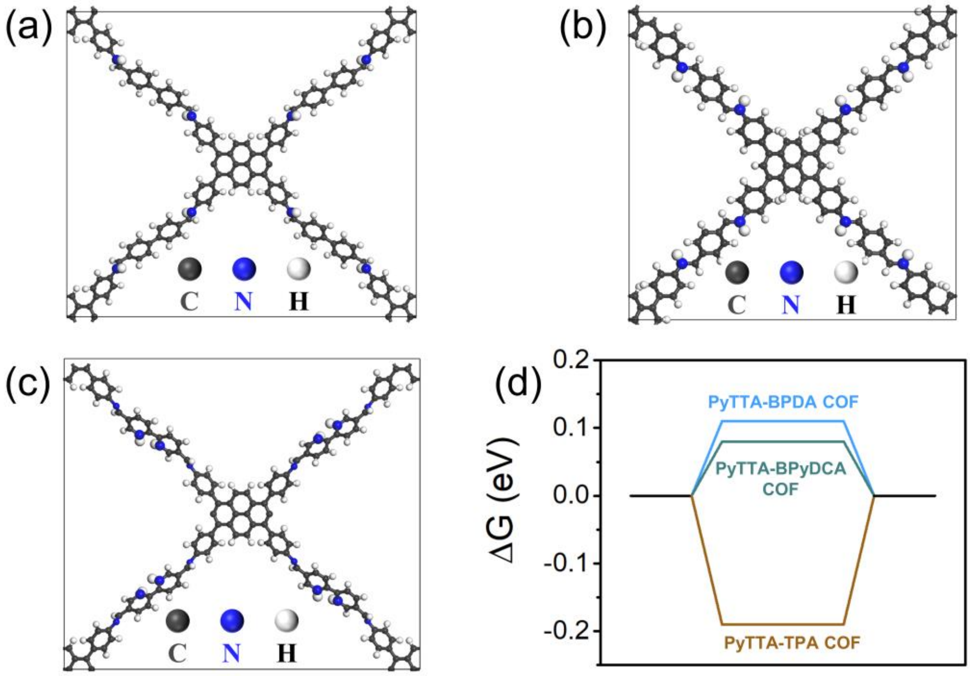

Supplementary Figure 43. DFT calculation results on COF film electrodes. (a–c) The atomic structures of PyTTA-BPDA COF, PyTTA-TPA COF, and PyTTA-BPyDCA COF, respectively. The N sites chosen for calculating the H adsorption free energy changes are shown in larger spheres. (d) Calculated free energy changes for HER of COF film.

The Gibbs free energy change (ΔG) was calculated to reveal the HER capacity of PyTTA-BPDA COF, PyTTA-TPA COF, and PyTTA-BPyDCA COF[15]. First principles density functional theory calculations were performed to obtain the free energy change for the HER of PyTTA-TPA, PyTTA-BPDA and PyTTA-BPyDCA COFs by the Vienna ab initio simulation Package (VASP) [16,17]. The interaction between valence electrons and ionic cores was dealt by the projected augmented wave (PAW) method[18]. The exchange-correlation potential was described by the Perdew-Burke-Ernzerhof (PBE) generalized gradient approximation (GGA) functional[19]. The DFT-D2 of Grimme method was employed for the correction of the weak van der Waals interaction in the COF systems[20]. The energy cutoff for the plane wave basis is set to be 400 eV. A k-point mesh with a distance of 0.03 $Å^{-1}$ between neighboring K points in the Monkhorst-Pack scheme was set[21]. All the structures were optimized until the force on each atom is less than 0.01 eV/Å with an energy convergence of 10^{-4} eV. The free energy change for HER is calculated by the same method as that in previous studies[22].

References

1. C.-Z. Guan, D. Wang, L.-J. Wan. *Chem. Commun.* **2012**, 48, 2943.

2. C. Lu, Y.-P. Mo, Y. Hong, T. Chen*, Z.-Y. Yang, L.-J. Wan, D. Wang. *J. Am. Chem. Soc.* **2020**, 142, 14350–14356.

3. W. Jiang, D. Peng, W. -R. Cui, R. -P. Liang, J. -D. Qiu. *ACS Omega* **2020**, 5, 32002.

4. Z.Wang, Q. Yu, Y. Huang, H. An, Y. Zhao, Y. Feng, X. Li, X. Shi, J. Liang, F. Pan, P. Cheng, Y. Chen, S. Ma, Z. Zhang. *ACS Cent Sci.* **2019**, 5, 1352.

5. A. P. Thompson a, H. M. Aktulga, R. Berger, D. S. Bolintineanu, W. M. Brown, P. S. Crozier, P. J. in 't Veld, A. Kohlmeyer, S. G. Moore, T. D. Nguyen, R. Shan, M. J. Stevens, J. Tranchida, C. Trott, S. J. Plimpton. *Comp. Phys. Comm.* 2022, 271, 108171.

6. L. Liu, Y. Liu, S. V. Zybin, H. Sun, and W.A. Goddard. *J. Phys. Chem. A.* **2011**, 115, 40, 11016.

7. L. Verlet. *Phys. Rev.* **1967**,159, 98.

8. S. Nosé. *J. Chem. Phys.***1984**, 81, 511.

9. W.-G. Hoover. *Phys. Rev. A.* **1985**, 31, 1695.

10. J. Guo, Y. Xu, S. Jin, L. Chen, T. Kaji, Y. Honsho, M. A. Addicoat, J. Kim, A. Saeki, H. Ihee, S. Seki, S. Irle, M. Hiramoto, J. Gao, D. Jiang. *Nat. Commun.* **2013**, 4, 2736.

11. T. Wu, A.R. Sheu, Y. Chen. *Macromolecules* **2004**, 37, 725.

12. W. Kutner, K. Noworyta, G.R. Deviprasad, F. Dsouza. *J. Electrochem. Soc.* **2000**, 147, 2647.

13. G. Wang, W. Huang, N. D. Eastham, S. Fabiano, E. F. Manley, L. Zeng, B. Wang, X. Zhang, Z. Chen, R. Li, R. P. H. Chang, L. X. Chen, M. J. Bedzyk, F. S. Melkonyan, A. Facchetti, T. J. Marks, Proc. *Natl. Acad. Sci.* U.S.A. **2017**, 114, E10066.

14. B. Sun, C. -H. Zhu, Y. Liu, C. Wang, L. -J. Wan, D. Wang, *Chem. Mater.* **2017**, 29, 4367.

15. B. C. Patra, S.Khilari, R. N.Manna, S. Mondal, D. Pradhan , A. Pradhan , A. Bhaumik. *ACS Catal.* **2017**, 7, 6120.

16. G. Kresse, J. Hafner. *Phys. Rev. B.* **1993**, 48, 13115.

17. G. Kresse, J. Furthmüller. *Comput. Mater. Sci.* **1996**, 6, 15.

18. G. Kresse, D. Joubert. *Phys. Rev. B.* **1999**, 59, 1758.

19. J. P. Perdew, K. Burke, M. Ernzerhof. *Phys. Rev. Lett.* **1996**, 77, 3865.

20. S. Grimme. *J Comput Chem.* **2006**, 27, 1787.

21. H. -J. Monkhorst, J. -D. Pack. *Phys. Rev. B.* **1976**, 13, 5188

22. J. Yang, A. R. Mohmad, Y. Wang, R. Fullon, X. Song, F. Zhao, I. Bozkurt, M. Augustin, E. J. G. Santos, H. S. Shin, W. Zhang, D. Voiry, H. Y. Jeong, M. Chhowalla, *Nat. Mater.* **2019**, 18, 1309–1314.
